# Data Release: DNA barcodes of plant species collected for the Global Genome Initiative for Gardens Program, National Museum of Natural History, Smithsonian Institution

**DOI:** 10.3897/phytokeys.88.14607

**Published:** 2017-10-12

**Authors:** Jose D. Zúñiga, Morgan R. Gostel, Daniel G. Mulcahy, Katharine Barker, Asia Hill, Maryam Sedaghatpour, Samantha Q. Vo, Vicki A. Funk, Jonathan A. Coddington

**Affiliations:** 1 Global Genome Initiative, MRC-106, National Museum of Natural History, Smithsonian Institution, P.O. Box 37012 Washington, DC 20013-7012 U.S.A.; 2 Department of Botany, MRC-166, National Museum of Natural History, Smithsonian Institution, P.O. Box 37012 Washington, DC 20013-7012 U.S.A.; 3 College of Education, North Carolina A&T University, 1601 East Market Street, Greensboro, N.C. 27411 U.S.A.; 4 University Herbarium and Department of Integrative Biology, University of California, 1001 Valley Life Sciences Building #2465, Berkeley, CA 94720 U.S.A.; 5 Environmental Science and Policy, George Mason University, 4400 University Drive, Fairfax, VA 22030 U.S.A.

**Keywords:** DNA barcoding, GenBank, land plants

## Abstract

The Global Genome Initiative has sequenced and released 1961 DNA barcodes for genetic samples obtained as part of the Global Genome Initiative for Gardens Program. The dataset includes barcodes for 29 plant families and 309 genera that did not have sequences flagged as barcodes in GenBank and sequences from officially recognized barcoding genetic markers meet the data standard of the Consortium for the Barcode of Life. The genetic samples were deposited in the Smithsonian Institution’s National Museum of Natural History Biorepository and their records were made public through the Global Genome Biodiversity Network’s portal. The DNA barcodes are now available on GenBank.

## Introduction

The Global Genome Initiative (GGI) is a Smithsonian Institution program to collect, organize, share, and study genomic samples of non-human species. The mission of GGI is to preserve and understand Earth’s genomic biodiversity. In pursuit of this mission, GGI aims to collect and preserve genome-quality tissue samples from all major lineages of life on Earth; foster biodiversity genomics research by generating DNA barcodes for dark taxa (i.e., those with no genetic data in online repositories); and promote the use of new technologies to study genomics across the tree of life. GGI supports the Global Genome Biodiversity Network (GGBN), an international network of institutions interested in the preservation of non-human genomic samples (Seberg et al. 2016). Members of GGBN can make their DNA and tissue collections discoverable on GGBN’s data portal (http://www.ggbn.org/ggbn_portal/), ensuring transparent access and visibility to the genetic resources to the research community.

The Global Genome Initiative for Gardens Program (GGI-Gardens) is a GGI-funded effort to collect and preserve genetic material from the plant Tree of Life that is not yet represented in any of GGBN’s partner institutions, and that are currently found in living collections around the globe. In its first phase, the program targeted living plant collections in the Washington, DC area and collected more than 1,800 genome-quality tissues from 209 families, 1007 genera and 1631 species. Moving forward, GGI-Gardens is focused on expanding its partnerships internationally to continue sample and preserve genomic biodiversity from all families and genera, and, potentially, species of plants on Earth.

The genetic samples collected to date have been deposited in the Smithsonian Institution’s National Museum of Natural History Biorepository (http://naturalhistory.si.edu/rc/biorepository) and are available upon request to researchers across the globe (regulations on sampling leaf material can be found here). All corresponding specimen vouchers have been accessioned in the United States National Herbarium (US) or other recognized, partner herbaria. The GGI-Gardens protocol (Gostel et al. 2016) and US National Herbarium best practices (Funk et al. 2017) have been published to facilitate the establishment of voucher programs at partner institutions.

GGI’s barcoding strategy data-mines GenBank to detect taxonomic groups that do not have sequences flagged as barcodes, thus allowing GGI to focus sequencing efforts on lineages that are not represented in this repository. Using this method, GGI selected more than 500 plant genera from GGI-Gardens collections and generated sequences for four genetic markers according to the DNA barcode data standard (Consortium for the Barcode of Life 2005). As a result, all sequences from officially accepted barcoding regions (two of the four markers targeted, see below) have been labeled with the keyword “BARCODE” in GenBank. All samples were determined at least to genus by the time of publication of this release paper by staff at the living collection where they were collected. Our intentions are to make these data publicly available, to contribute to the DNA Barcode library to assist further research, and to make the presence of these genomic-quality tissues known and available for the academic community for genomic research and education purposes via a documented application process. All DNA barcode sequences were submitted to GenBank as part of the GGI-Gardens BioProject (ID: PRJNA389125), which is included in Global Genome Initiative’s DNA Barcoding umbrella BioProject (ID: PRJNA384793).

## Data resources and contents of the dataset

Data are deposited in GenBank under accession numbers MF348326-MF350286 (see supplementary file 1 for the full list of accession numbers). A total of 1961 sequences have been submitted to GenBank representing 160 families and 521 plant genera, including 29 families and 309 genera that previously did not have sequences flagged as barcodes in this data repository. Two of the four genetic markers sequenced, *rbcL* and *matK*, have been officially recognized as barcoding regions for land plants (CBOL Plant Working Group 2009). The other two loci targeted in this study, the nuclear ribosomal internal transcribed spacer (nrITS) and the plastid *psbA-trnH* intergenic spacer, are commonly used for barcoding in angiosperms (Kress et al. 2005, Kress and Erickson 2009, Hollingsworth et al. 2016).

## Supplementary materials

Supplementary material 1 List of samples collected for the Global Genome Initiative for Gardens project selected for DNA barcoding, with GenBank accession numbers and genetic sample identification numbers. All the sequences are included in the GGI-Gardens BioProject. Data type: Microsoft Word Document (.docx) Explanation note: List of samples collected for the Global Genome Initiative for Gardens project selected for DNA barcoding, with GenBank accession numbers and genetic sample identification numbers. This dataset is made available under the Open Database License (http://opendatacommons.org/licenses/odbl/1.0/). The Open Database License (ODbL) is a license agreement intended to allow users to freely share, modify, and use this Dataset while maintaining this same freedom for others, provided that the original source and author(s) are credited.Jose D. Zúñiga, Morgan R. Gostel, Daniel G. Mulcahy, Katharine Barker, Asia Hill, Maryam Sedaghatpour, Samantha Q. Vo, Vicki A. Funk, Jonathan A. Coddington
