# Supplementary material for: Data Release: DNA barcodes of plant species collected for the Global Genome Initiative for Gardens Program, National Museum of Natural History, Smithsonian Institution
Source: PhytoKeys. 2017 Oct 12;(88):119–22. doi: 10.3897/phytokeys.88.14607 (PMC5672136; doi:10.3897/phytokeys.88.14607)
Supplement: Supplementary material 1 — List of samples collected for the Global Genome Initiative for Gardens project selected for DNA barcoding, with GenBank accession numbers and genetic sample identification numbers. All the sequences are included in the GGI-Gardens BioProject. [file phytokeys-88-119-s001.doc]

Supplementary File 1: List of samples collected for the Global Genome Initiative for Gardens project selected for DNA barcoding, with GenBank accession numbers and genetic sample identification numbers. All the sequences are included in the [GGI-Gardens BioProject](https://www.ncbi.nlm.nih.gov/bioproject/389125).

| **NMNH Biorepository**  **Tissue ID** | **Collector Number** | **Family** | **Genus** | **Species** | ***rbcL*** | ***matK*** | ***psbA-trnH*** | **ITS** |
| --- | --- | --- | --- | --- | --- | --- | --- | --- |
| AD7LH75 | [Van Neste 770](http://n2t.net/ark:/65665/3623c391a-a113-45c9-819e-d08ac0c880fa) | Acanthaceae | *Acanthus* | *spinosus* | MF349678 | MF350143 | MF348488 |  |
| AG2KK73 | [Marcelli 8](http://n2t.net/ark:/65665/3e01ae452-267a-4ff4-9763-60bd3676e583) | Acanthaceae | *Andrographis* | *paniculata* | MF349365 | MF349897 | MF348796 |  |
| AD5JP51 | [Van Neste 553](http://n2t.net/ark:/65665/3cfbc6f46-947d-4e31-babf-4243df987c06) | Acanthaceae | *Anisacanthus* | *quadrifidus* | MF349712 | MF350158 | MF348455 |  |
| AD3HI87 | [Van Neste 281](http://n2t.net/ark:/65665/3d5e68573-ab5f-4620-9018-a0c477847636) | Acanthaceae | *Aphelandra* | *aurantiaca* | MF349506 | MF350014 | MF348669 |  |
| AD3HI64 | [Van Neste 258](http://n2t.net/ark:/65665/33343044f-0379-4d5c-8c98-ee0356f5ab31) | Acanthaceae | *Hemigraphis* | *alternata* | MF349495 | MF350007 | MF348678 |  |
| AD0EE86 | [Van Neste 364](http://n2t.net/ark:/65665/37fd7161f-2962-4c57-8451-88c217641d0f) | Acanthaceae | *Justicia* | *americana* | MF349778 | MF350205 | MF348382 |  |
| AD3HJ70 | [Van Neste 364](http://n2t.net/ark:/65665/37fd7161f-2962-4c57-8451-88c217641d0f) | Acanthaceae | *Justicia* | *americana* | MF349471 | MF349994 | MF348700 |  |
| AD5JP66 | [Van Neste 568](http://n2t.net/ark:/65665/30bdb56a0-960b-45c1-9564-7fe45f39da11) | Acanthaceae | *Pachystachys* | *lutea* | MF349740 | MF349830 | MF348426 |  |
| AD3HL10 | [Van Neste 506](http://n2t.net/ark:/65665/3fa059c63-02e9-42bc-a9d3-05054dca254c) | Acanthaceae | *Ruellia* | *humilis* | MF349499 | MF350010 | MF348675 | MF349071 |
| AD5JT44 | [Van Neste 672](http://n2t.net/ark:/65665/3e00f7e56-2988-4966-b98c-dd2924cdcfa4) | Acanthaceae | *Ruellia* | *simplex* | MF349704 | MF350155 | MF348463 | MF348923 |
| AG2KL21 | [Sedaghatpour 2016-64](http://n2t.net/ark:/65665/3d6fdbbec-cc66-46b2-a30a-32c37903f71e) | Acanthaceae | *Strobilanthes* | *gossypina* | MF349322 | MF349863 | MF348829 |  |
| AC9DD66 | [Van Neste 538](http://n2t.net/ark:/65665/3a83f6747-296c-4423-9672-7c2feb79af1d) | Aceraceae | *Acer* | *henryi* | MF349813 | MF350233 | MF348336 | MF348850 |
| AG2KM37 | [Sedaghatpour 2016-84](http://n2t.net/ark:/65665/38fdb4077-d21f-4254-9483-9ffac5f517e7) | Agavaceae | *Manfreda* | *virginica* | MF349339 | MF349877 |  | MF349179 |
| AD5JS92 | [Van Neste 682](http://n2t.net/ark:/65665/37ab7dabf-4e85-4e37-ba12-9d1042b970c7) | Aizoaceae | *Conophytum* | *violaciflorum* | MF349695 |  | MF348473 | MF348932 |
| AD5JS93 | [Van Neste 683](http://n2t.net/ark:/65665/34de6b77f-a016-4513-99fe-74959dd5069b) | Aizoaceae | *Fenestraria* | *rhopalophylla* | MF349270 |  |  |  |
| AD5JS94 | [Van Neste 684](http://n2t.net/ark:/65665/3eb54b4d1-8468-447b-9955-c3e21d220a49) | Aizoaceae | *Pleiospilos* | *compactus* | MF349684 |  | MF348483 |  |
| AD0EF12 | [Van Neste 412](http://n2t.net/ark:/65665/3cdda5437-0ac6-454b-b2a7-10af0b87a5f4) | Alismataceae | *Alisma* | *subcordatum* | MF349289 | MF350279 |  |  |
| AD0EF12 | [Van Neste 412](http://n2t.net/ark:/65665/3cdda5437-0ac6-454b-b2a7-10af0b87a5f4) | Alismataceae | *Alisma* | *subcordatum* |  |  | MF348402 |  |
| AD7LH23 | [Van Neste 746](http://n2t.net/ark:/65665/3e11d505b-9e94-46b4-9410-0d95eec13c22) | Altingiaceae | *Liquidambar* | *styraciflua* | MF349279 |  | MF348497 |  |
| AC9DD17 | [Van Neste 216](http://n2t.net/ark:/65665/3f261ce9c-5e0c-4077-890d-a79c126fc7c0) | Amaranthaceae | *Nototrichium* | *sandwicense* | MF349805 | MF350227 |  | MF348854 |
| AD0ED93 | [Van Neste 286](http://n2t.net/ark:/65665/34a48470f-2853-479d-90ae-d3381d5a4016) | Amaryllidaceae | *Clivia* | *miniata* | MF349780 | MF350206 | MF348379 |  |
| AG2KL29 | [Sedaghatpour 2016-68](http://n2t.net/ark:/65665/30bf6f2b8-d7c4-4214-b9e6-6379f6dd57b1) | Amaryllidaceae | *Habranthus* | *robustus* | MF349374 | MF349905 | MF348790 | MF349159 |
| AD7LL49 | [Sedaghatpour 2016-36](http://n2t.net/ark:/65665/38801bcb3-d800-4533-a3a3-946e9e1047d4) | Amaryllidaceae | *Ipheion* | *uniflorum* | MF349400 | MF349927 | MF348773 | MF349140 |
| AD7LM24 | [Faulconer 34](http://n2t.net/ark:/65665/342b3e58e-7f76-46f4-937f-8183f08b04f2) | Amaryllidaceae | *Leucojum* | *aestivum* | MF349433 | MF349959 | MF348739 | MF349115 |
| AD7LM69 | [Sedaghatpour 2016-54](http://n2t.net/ark:/65665/350c0c93e-03c6-49f7-8de1-2ca405d57fd1) | Amaryllidaceae | *Lycoris* | spp. | MF349227 | MF350247 | MF348757 |  |
| AG2KM39 | [Sedaghatpour 2016-85](http://n2t.net/ark:/65665/36181bed3-138e-4700-bd81-6688ba993606) | Amaryllidaceae | *Narcisus* | *poeticus* | MF349327 | MF349869 | MF348822 | MF349189 |
| AD7LJ07 | [Gostel 156](http://n2t.net/ark:/65665/3626981f9-9370-4236-8ce4-f525fa3082e0) | Amaryllidaceae | *Polianthes* | *howardii* | MF349426 | MF349954 |  |  |
| AG2KL27 | [Sedaghatpour 2016-67](http://n2t.net/ark:/65665/369f7ebc4-57cb-4539-8ea4-191a13b62d5c) | Amaryllidaceae | *Zephyranthes* | *robust* | MF349383 | MF349912 | MF348785 | MF349153 |
| AG2KM03 | [Marcelli 15](http://n2t.net/ark:/65665/333faf49c-0d0f-4b8c-b741-208f4c704d4f) | Anacardiaceae | *Cotinus* | *coggygria* | MF349350 | MF349885 | MF348807 | MF349173 |
| AD0EF25 | [Van Neste 396](http://n2t.net/ark:/65665/3e2c25053-710c-46f4-a40e-173eab9d462e) | Anacardiaceae | *Rhus* | *glabra* | MF349300 | MF349825 | MF348360 |  |
| AD3HK29 | [Van Neste 423](http://n2t.net/ark:/65665/318eff1d5-b6b7-4661-89cf-dc0ff689ee6b) | Annonaceae | *Asimina* | *triloba* | MF349596 | MF350078 | MF348576 |  |
| AG2KL89 | [Sedaghatpour 2016- 72](http://n2t.net/ark:/65665/3f2591427-c833-4f41-a9c4-40fb6786122d) | Annonaceae | *Cananga* | *odorata* | MF349372 | MF349903 | MF348792 | MF349161 |
| AD5JS73 | [Van Neste 626](http://n2t.net/ark:/65665/33182d3a1-8727-4df9-95cf-b9738ddd0019) | Apiaceae | *Cicuta* | *maculata* | MF349747 | MF350179 | MF348419 | MF348895 |
| AC9DD33 | [Van Neste 232](http://n2t.net/ark:/65665/392e541c8-e096-4115-bc7f-e0030ce2c11c) | Apiaceae | *Eryngium* | *foetidum* | MF349304 | MF350226 | MF348348 | MF348855 |
| AD3HI38 | [Van Neste 232](http://n2t.net/ark:/65665/392e541c8-e096-4115-bc7f-e0030ce2c11c) | Apiaceae | *Eryngium* | *foetidum* | MF349509 | MF350016 | MF348667 | MF349066 |
| AD3HK81 | [Van Neste 477](http://n2t.net/ark:/65665/325bf98f4-9d95-433f-9b21-9aa4d377ddef) | Apiaceae | *Sanicula* | *canadensis* | MF349559 | MF350053 | MF348618 | MF349027 |
| AD7LM85 | [Vo 80](http://n2t.net/ark:/65665/38dd88e4d-2ba1-4b74-8676-381ef75c657a) | Apiaceae | *Zizia* | *aurea* | MF349413 | MF349941 | MF348758 | MF349128 |
| AD3HL95 | [Van Neste 573](http://n2t.net/ark:/65665/399aa85d7-0b36-4765-90c1-453015e8b438) | Apocynaceae | *Allamanda* | *blanchetii* | MF349792 | MF350217 | MF348366 | MF348867 |
| AG2KK39 | [Gostel 213](http://n2t.net/ark:/65665/32c37f629-c8d2-42c4-ae4b-c7316c408aef) | Apocynaceae | *Plumeria* | *obtusa* | MF349346 |  |  |  |
| AG2KK35 | [Gostel 211](http://n2t.net/ark:/65665/3f202dee0-13c4-4bcb-9dcb-7c0b4e2ecf5c) | Apocynaceae | *Strophanthus* | *preussii* | MF349367 | MF349898 | MF348795 |  |
| AD3HJ12 | [Van Neste 306](http://n2t.net/ark:/65665/3b1f2a1ef-d89d-48e9-9539-3d4a06418797) | Aquifoliaceae | *Ilex* | *verticillata* | MF349474 | MF349996 | MF348697 | MF349080 |
| AD3HI88 | [Van Neste 282](http://n2t.net/ark:/65665/3f70688b1-2b84-49c0-b71d-f6a8fcd9aae4) | Araceae | *Aglaonema* | *commutatum* | MF349494 | MF350006 | MF348679 |  |
| AD3HI83 | [Van Neste 277](http://n2t.net/ark:/65665/38b85e7b9-342d-473d-b7be-11805f8f121a) | Araceae | *Aglaonema* | *crispum* | MF349528 | MF350029 | MF348650 |  |
| AG2KL25 | [Sedaghatpour 2016-66](http://n2t.net/ark:/65665/30395f2de-f9cf-4474-8854-7a47e09267fa) | Araceae | *Anchomanes* | *dalzielii* | MF349312 | MF349857 | MF348838 |  |
| AD3HI86 | [Van Neste 280](http://n2t.net/ark:/65665/33a7cf7c7-348f-4413-aa7b-fafebc907c07) | Araceae | *Anthurium* | *bakeri* | MF349516 | MF350022 | MF348659 |  |
| AD7LI27 | [Van Neste 802](http://n2t.net/ark:/65665/3b2d54113-436f-42a6-9397-33f58bbc9236) | Araceae | *Anthurium* | *polyschistum* | MF349657 | MF350126 | MF348512 |  |
| AD7LI31 | [Van Neste 806](http://n2t.net/ark:/65665/3ecaa88ce-d22f-4c35-b4eb-dbc343ac0eb7) | Araceae | *Anthurium* | *vittariifolium* | MF349273 | MF350110 | MF348531 |  |
| AD3HK50 | [Van Neste 444](http://n2t.net/ark:/65665/3f57e5123-83aa-4a49-a46f-43482061e630) | Araceae | *Arisaema* | *triphyllum* | MF349545 | MF350040 | MF348635 |  |
| AD3HK86 | [Van Neste 482](http://n2t.net/ark:/65665/3b915dfa2-dd50-44e0-87f7-acf3d0c89946) | Araceae | *Arum* | *italicum* | MF349542 | MF350038 | MF348637 | MF349044 |
| AD7LI23 | [Van Neste 798](http://n2t.net/ark:/65665/3b2ce80a2-db74-48eb-9d4b-40e62d86c6e5) | Araceae | *Gonatopus* | *boivinii* | MF349667 | MF350134 | MF348501 |  |
| AB6QQ08 | [Van Neste 108](http://n2t.net/ark:/65665/311e9e372-38d5-4fbb-a7a4-b5303e97fe6e) | Araceae | *Spathiphyllum* | *cannifolium* | MF349733 | MF350170 |  | MF348904 |
| AD5JS54 | [Van Neste 607](http://n2t.net/ark:/65665/376867bfd-e35d-41a5-b8dc-329ffe9b208d) | Araceae | *Symplocarpus* | *foetidus* | MF349749 | MF350181 | MF348417 |  |
| AD7LJ05 | [Gostel 154](http://n2t.net/ark:/65665/398a39217-a757-4aef-9bf0-12635c87d7aa) | Araliaceae | *Eleutherococcus* | *senticosus* | MF349434 | MF349961 | MF348735 | MF349112 |
| AD7LN07 | [Faulconer 51](http://n2t.net/ark:/65665/345a3ad29-7511-448a-942f-b45a6e4fa61a) | Araliaceae | *Fatsia* | *japonica* | MF349455 | MF349983 |  | MF349096 |
| AD5JQ08 | [Kelloff 1625](http://n2t.net/ark:/65665/3ea46af28-c567-44e4-84be-32dd8772223f) | Araliaceae | *Hydrocotyle* | *umbellata* | MF349776 | MF350202 | MF348386 |  |
| AG2KK25 | [Gostel 206](http://n2t.net/ark:/65665/3106b5873-3a3a-47f0-9a28-9482d6acd26f) | Araucariaceae | *Araucaria* | *araucana* | MF349336 |  | MF348814 | MF349182 |
| AD3HI16 | [Van Neste 210](http://n2t.net/ark:/65665/3072eb3e8-172e-44bc-86bb-73207d1443cc) | Arecaceae | *Ptychosperma* | *burretianum* | MF349498 | MF350267 | MF348676 |  |
| AB6QQ30 | [Van Neste 131](http://n2t.net/ark:/65665/3263a48b2-d8a0-42e3-a6f3-b856d8aa499c) | Arecaceae | *Rhapidophyllum* | *hystrix* | MF349288 | MF350189 |  |  |
| AG2KK41 | [Gostel 214](http://n2t.net/ark:/65665/302e01c58-cca7-4012-aea6-3d9654678726) | Aristolochiaceae | *Aristolochia* | *fimbriata* | MF349335 | MF349875 | MF348815 | MF349183 |
| AD7LM91 | [Faulconer 43](http://n2t.net/ark:/65665/353279487-411c-49ef-a0d2-9242a7343b48) | Asclepiadaceae | *Cynanchum* | *ascyrifolium* | MF349456 | MF349984 | MF348711 |  |
| AG2KL11 | [Sedaghatpour 2016-59](http://n2t.net/ark:/65665/35a5006b2-664f-4204-89b5-6814a3ed1d42) | Asparagaceae | *Albuca* | *spiralis* | MF349375 | MF349906 |  |  |
| AG2KL75 | [Ingram 16](http://n2t.net/ark:/65665/33725d5ab-bc65-44b2-92e8-576f32481ab2) | Asparagaceae | *Disporopsis* | *pernyi* | MF349329 | MF349871 | MF348820 |  |
| AD0EG82 | [Van Neste 548](http://n2t.net/ark:/65665/3fc64e8d2-945a-48f6-b81c-bdfa035a5052) | Asparagaceae | *Hesperaloe* | *campanulata* | MF349815 | MF350234 | MF348334 |  |
| AD3HJ56 | [Van Neste 350](http://n2t.net/ark:/65665/3806cb987-06fb-4c75-99d1-a75c84015d92) | Asparagaceae | *Hosta* | *sieboldiana* | MF349480 | MF349999 | MF348690 |  |
| AD7LN19 | [Faulconer 57](http://n2t.net/ark:/65665/3dff33fdf-eb03-42ff-953b-029936fbf4e9) | Asparagaceae | *Hyacinthus* | spp. | MF349236 | MF349930 | MF348770 |  |
| AD7LM28 | [Faulconer 36](http://n2t.net/ark:/65665/34d32d3f0-6e1e-471b-a843-bde066d038fb) | Asparagaceae | *Muscari* | *armeniacum* | MF349424 | MF349951 | MF348747 |  |
| AG2KL69 | [Ingram 13](http://n2t.net/ark:/65665/3b37594d8-8ac3-43aa-ad24-c90680280d98) | Asparagaceae | *Ophiopogon* | *planiscapus* | MF349361 | MF349894 | MF348798 |  |
| AB6QQ11 | [Van Neste 111](http://n2t.net/ark:/65665/3f4ca9c00-5323-4c2a-bf74-31f25814f90e) | Asparagaceae | *Peliosanthes* | *teta* | MF349724 |  | MF348442 |  |
| AG2KM09 | [Marcelli 18](http://n2t.net/ark:/65665/329fabe80-7072-4735-9ad9-4a1efd294abc) | Asparagaceae | *Ruscus* | *aculeatus* | MF349328 | MF349870 | MF348821 |  |
| AG2KK33 | [Gostel 210](http://n2t.net/ark:/65665/3432e3919-7c51-4c12-8404-b8a9371ec6ee) | Asparagaceae | *Sansevieria* | *masoniana* | MF349377 | MF349909 | MF348788 |  |
| AG2KK01 | [Gostel 194](http://n2t.net/ark:/65665/3566da0ce-e576-41e6-a206-ece1277814c9) | Asparagaceae | *Triteleia* | *laxa* | MF349348 | MF349882 |  |  |
| AD3HI21 | [Van Neste 215](http://n2t.net/ark:/65665/3b4bf5bca-533c-46f5-957b-d821eb03d1c1) | Aspleniaceae | *Asplenium* | *kaulfussii* | MF349250 |  | MF348664 |  |
| AD3HL60 | [Kelloff 1577](http://n2t.net/ark:/65665/33ad0d75b-ae18-454b-8a2a-3b66e11487a7) | Aspleniaceae | *Asplenium* | *platyneuron* | MF349464 |  | MF348692 |  |
| AD3HJ35 | [Van Neste 329](http://n2t.net/ark:/65665/3cbda254f-441b-4530-ae4a-6fe1219df969) | Asteraceae | *Achillea* | *sibirica* | MF349503 | MF350012 | MF348671 |  |
| AD0EE44 | [Van Neste 332](http://n2t.net/ark:/65665/30c446054-e914-4779-8723-0147396b91b0) | Asteraceae | *Anthemis* | spp. | MF349234 | MF350284 | MF348400 |  |
| AD3HJ38 | [Van Neste 332](http://n2t.net/ark:/65665/30c446054-e914-4779-8723-0147396b91b0) | Asteraceae | *Anthemis* | spp. | MF349210 | MF350000 | MF348689 |  |
| AD3HI31 | [Van Neste 225](http://n2t.net/ark:/65665/378d37a23-989b-4f0b-87bb-41028488a82d) | Asteraceae | *Aspilia* | *mossambicensis* | MF349538 | MF350036 | MF348640 |  |
| AG2KK43 | [Gostel 215](http://n2t.net/ark:/65665/32a832adb-1951-4140-9233-d6e96aafe76d) | Asteraceae | *Aster* | spp. | MF349212 | MF349866 | MF348825 | MF349193 |
| AD3HL09 | [Van Neste 505](http://n2t.net/ark:/65665/36b44db0e-56e4-42e1-9c66-cdbcc3b98c8f) | Asteraceae | *Berlandiera* | *texana* | MF349511 | MF350018 | MF348665 | MF349064 |
| AD3HK70 | [Van Neste 466](http://n2t.net/ark:/65665/397ac6c11-2b42-4d16-9998-85148e383043) | Asteraceae | *Carduus* | spp. | MF349218 | MF350045 | MF348627 | MF349037 |
| AD5JQ74 | [Kelloff 1613](http://n2t.net/ark:/65665/3373a1f48-c268-4779-9af5-94590dde3773) | Asteraceae | *Carphephorus* | *bellidifolius* | MF349262 |  | MF348592 | MF349006 |
| AD5JQ50 | [Kelloff 1589](http://n2t.net/ark:/65665/35c2652db-287a-47c5-8689-ad04cca75f28) | Asteraceae | *Centaurea* | *stoebe* | MF349573 | MF350063 | MF348601 | MF349012 |
| AG2KK87 | [Ingram 1](http://n2t.net/ark:/65665/3fae85c19-4a1e-4259-8d50-5f28c9b164d8) | Asteraceae | *Chrysogonum* | *virginianum* | MF349314 | MF349858 | MF348836 | MF349203 |
| AD5JQ73 | [Kelloff 1612](http://n2t.net/ark:/65665/36a4b182e-d461-4511-ae68-ad256635b8e5) | Asteraceae | *Chrysopsis* | *graminifolia* | MF349263 | MF350074 |  |  |
| AD7LH12 | [Van Neste 735](http://n2t.net/ark:/65665/389b70166-ffb9-43cf-87b9-2833f67011f6) | Asteraceae | *Cichorium* | spp. | MF349222 | MF350121 | MF348518 |  |
| AD5JQ86 | [Kelloff 1627](http://n2t.net/ark:/65665/3baeb6ba3-3536-4efa-bb48-a2fd3f2926eb) | Asteraceae | *Conoclinium* | *coelestinum* | MF349571 | MF350271 | MF348603 | MF349014 |
| AD5JQ58 | [Kelloff 1597](http://n2t.net/ark:/65665/3c927ce19-92ae-4366-b08b-07c75972990d) | Asteraceae | *Conyza* | *canadensis* | MF349541 | MF350068 | MF348591 | MF349005 |
| AD3HL07 | [Van Neste 503](http://n2t.net/ark:/65665/3b0929729-3a3e-41c3-9b84-7fbe9d799aad) | Asteraceae | *Coreopsis* | *pubescens* | MF349532 | MF350031 | MF348647 |  |
| AD3HL05 | [Van Neste 501](http://n2t.net/ark:/65665/303f6af86-53bb-4925-a869-5e266300571e) | Asteraceae | *Coreopsis* | *tripteris* | MF349540 | MF350037 | MF348638 | MF349045 |
| AD5JT23 | [Van Neste 650](http://n2t.net/ark:/65665/34d79a78e-c4d5-47d0-8425-9b81afeaae27) | Asteraceae | *Cynara* | *cardunculus* | MF349717 | MF350159 | MF348450 |  |
| AD0EE57 | [Van Neste 341](http://n2t.net/ark:/65665/308aa8ac6-d427-4a7d-9bca-499d27cb8203) | Asteraceae | *Echinacea* | *pallida* | MF349294 | MF349827 | MF348389 |  |
| AD3HJ47 | [Van Neste 341](http://n2t.net/ark:/65665/308aa8ac6-d427-4a7d-9bca-499d27cb8203) | Asteraceae | *Echinacea* | *pallida* | MF349252 |  | MF348644 |  |
| AD0EG44 | [Kelloff 1567](http://n2t.net/ark:/65665/3013c3d5a-34cb-4376-b068-2732ffc749e7) | Asteraceae | *Echinacea* | *purpurea* | MF349209 |  | MF348385 |  |
| AD3HL50 | [Kelloff 1567](http://n2t.net/ark:/65665/3013c3d5a-34cb-4376-b068-2732ffc749e7) | Asteraceae | *Echinacea* | *purpurea* | MF349582 | MF349847 | MF348589 | MF349003 |
| AD5JS68 | [Van Neste 621](http://n2t.net/ark:/65665/355125cf0-ad47-4922-909f-b1834afd4424) | Asteraceae | *Elephantopus* | *carolinianus* | MF349719 | MF349834 |  | MF348914 |
| AG2KK77 | [Marcelli 10](http://n2t.net/ark:/65665/34a3b71a6-ed0b-419a-9034-8f78b24a31cb) | Asteraceae | *Engelmannia* | *peristenia* | MF349355 | MF349890 | MF348803 | MF349171 |
| AD3HL53 | [Kelloff 1570](http://n2t.net/ark:/65665/366f4b063-7c5e-45ca-a0bd-080c388da731) | Asteraceae | *Erigeron* | *annuus* | MF349557 | MF350051 | MF348620 | MF349029 |
| AD0EE55 | [Van Neste 339](http://n2t.net/ark:/65665/313f06847-ae81-4050-baf4-416fe893e3b9) | Asteraceae | *Erigeron* | spp. | MF349229 |  | MF348349 |  |
| AD3HJ45 | [Van Neste 339](http://n2t.net/ark:/65665/313f06847-ae81-4050-baf4-416fe893e3b9) | Asteraceae | *Erigeron* | spp. | MF349216 | MF349849 | MF348704 | MF349088 |
| AD7LH34 | [Van Neste 757](http://n2t.net/ark:/65665/3348a6cb5-72f9-4fca-974b-fc2ca4d7e699) | Asteraceae | *Eupatorium* | spp. | MF349226 | MF350091 |  |  |
| AD7LH39 | [Van Neste 763](http://n2t.net/ark:/65665/399132922-71b0-4686-99f9-020c2b8335d5) | Asteraceae | *Eupatorium* | *torreyanum* | MF349650 | MF350120 | MF348520 | MF348960 |
| AD7LN65 | [Van Neste 724](http://n2t.net/ark:/65665/37d50dd1b-130c-4f80-9bcf-015c8de14466) | Asteraceae | *Gaillardia* | *pulchella* | MF349662 | MF350129 | MF348506 | MF348951 |
| AD7LN76 | [Van Neste 713](http://n2t.net/ark:/65665/34c647c77-5592-4e39-ad2e-4ecad6f3dbf3) | Asteraceae | *Helianthus* | *debilis* | MF349643 | MF350116 | MF348525 | MF348964 |
| AD3HK45 | [Van Neste 439](http://n2t.net/ark:/65665/31c75ac47-ea42-49af-a8fa-50a5a2507f78) | Asteraceae | *Helianthus* | *divaricatus* | MF349561 | MF350261 | MF348616 | MF349024 |
| AD3HL14 | [Van Neste 510](http://n2t.net/ark:/65665/3290118e4-bc2e-4f05-84a4-57d397bc2b59) | Asteraceae | *Helianthus* | *microcephalus* | MF349249 |  | MF348693 |  |
| AD0EE56 | [Van Neste 340](http://n2t.net/ark:/65665/33b2b1aa1-f740-46f3-9509-a5af8342e1ce) | Asteraceae | *Heliopsis* | *helianthoides* | MF349797 | MF350221 | MF348359 | MF348862 |
| AD7LH37 | [Van Neste 760](http://n2t.net/ark:/65665/35d45dac3-9336-441c-8f6f-d5403c5bf24c) | Asteraceae | *Heliopsis* | *helianthoides* | MF349669 | MF349843 | MF348498 | MF348947 |
| AD5JQ70 | [Kelloff 1609](http://n2t.net/ark:/65665/312e708f3-8a09-4e54-bf06-58968a10822d) | Asteraceae | *Hieracium* | *gronovii* | MF349600 | MF350081 | MF348573 | MF348990 |
| AD3HL63 | [Kelloff 1580](http://n2t.net/ark:/65665/36fc4df36-ea48-4318-9d2e-d8e5bee93775) | Asteraceae | *Hypochaeris* | *radicata* | MF349264 | MF350083 | MF348571 | MF348989 |
| AG2KK63 | [Marcelli 3](http://n2t.net/ark:/65665/32ef2ff95-371f-4e37-b2d6-be0281659c60) | Asteraceae | *Krigia* | *biflora* | MF349325 | MF349865 | MF348826 | MF349194 |
| AD3HK76 | [Van Neste 472](http://n2t.net/ark:/65665/367b2b025-a280-4e55-a958-8d2d3a37fe38) | Asteraceae | *Krigia* | spp. | MF349214 | MF350076 | MF348578 | MF348994 |
| AD3HK73 | [Van Neste 469](http://n2t.net/ark:/65665/346bfc1e3-ddc6-4d35-816b-e77003a27217) | Asteraceae | *Lactuca* | *canadensis* | MF349543 | MF350039 | MF348636 | MF349043 |
| AG2KK57 | [Vo 88](http://n2t.net/ark:/65665/382c68355-8bda-4dc8-bfc5-33f4f3d7d719) | Asteraceae | *Leucanthemum* | x *superbum* | MF349356 | MF350250 |  |  |
| AD5JT26 | [Van Neste 653](http://n2t.net/ark:/65665/31e62398e-76a0-4622-bb0a-fc2d9f09c90f) | Asteraceae | *Liatris* | *microcephala* | MF349705 | MF350156 | MF348462 | MF348922 |
| AD3HI18 | [Van Neste 212](http://n2t.net/ark:/65665/3ffb58521-a700-475a-acda-2835e750581a) | Asteraceae | *Lipochaeta* | *connata* | MF349533 | MF350032 | MF348646 |  |
| AD5JT09 | [Van Neste 699](http://n2t.net/ark:/65665/3661884f1-c07b-4f0d-9455-59dfade7404c) | Asteraceae | *Marshallia* | *graminifolia* | MF349664 | MF350131 | MF348504 |  |
| AD3HI15 | [Van Neste 209](http://n2t.net/ark:/65665/319a209d8-7a84-4a43-8b1e-68238f19f0bb) | Asteraceae | *Marshallia* | *mohrii* | MF349510 | MF350017 | MF348666 | MF349065 |
| AD7LL59 | [Sedaghatpour 2016-41](http://n2t.net/ark:/65665/361b00aab-7f31-45dd-ae7f-3fc540076901) | Asteraceae | *Packera* | *aurea* | MF349425 | MF349953 | MF348745 | MF349120 |
| AD3HK93 | [Van Neste 489](http://n2t.net/ark:/65665/35dc87ee4-26b8-42af-945c-381f3ebe7ad7) | Asteraceae | *Parthenium* | *integrifolium* | MF349593 | MF350272 | MF348579 | MF348995 |
| AD5JQ63 | [Kelloff 1602](http://n2t.net/ark:/65665/35a66aa60-fd76-44a0-9337-978172bdfb8a) | Asteraceae | *Pseudognaphalium* | *obtusifolium* | MF349256 | MF350050 |  | MF349031 |
| AD5JS84 | [Van Neste 637](http://n2t.net/ark:/65665/3a5738484-e2a3-4d87-9b40-d25a663efe89) | Asteraceae | *Ratibida* | *columnifera* | MF349706 | MF350278 | MF348461 | MF348921 |
| AD5JT38 | [Van Neste 665](http://n2t.net/ark:/65665/308b88ca0-a41b-469b-916d-63d394ca6bcf) | Asteraceae | *Ratibida* | *pinnata* | MF349735 | MF350172 | MF348432 |  |
| AD3HL13 | [Van Neste 509](http://n2t.net/ark:/65665/300d53b0f-94f5-468e-aae2-d7acef3f6c67) | Asteraceae | *Rudbeckia* | *scabrifolia* | MF349488 |  | MF348683 | MF349076 |
| AD5JS71 | [Van Neste 624](http://n2t.net/ark:/65665/3d4d58113-7d96-4a14-ac98-c15b9c40d646) | Asteraceae | *Rudbeckia* | *triloba* | MF349690 |  | MF348479 | MF348936 |
| AD5JS97 | [Van Neste 687](http://n2t.net/ark:/65665/38f2eb4ba-c485-43e6-a3fa-5cb78fc2c8a6) | Asteraceae | *Senecio* | *nyikensis* | MF349654 | MF350124 | MF348515 | MF348956 |
| AD3HL15 | [Van Neste 511](http://n2t.net/ark:/65665/34cb38eec-795b-47a7-9328-78cb40440d7c) | Asteraceae | *Silphium* | *albiflorum* | MF349470 |  | MF348701 | MF349083 |
| AD7LH96 | [Van Neste 791](http://n2t.net/ark:/65665/399b14e93-6ce6-4e5f-9f6f-39741392de7b) | Asteraceae | *Silphium* | *compositum* | MF349274 | MF349844 |  | MF348967 |
| AD0EE54 | [Van Neste 338](http://n2t.net/ark:/65665/31006c933-5e53-4427-a27a-72e6c0431ce2) | Asteraceae | *Silphium* | *laciniatum* | MF349298 | MF349823 | MF348371 |  |
| AG2KK61 | [Marcelli 2](http://n2t.net/ark:/65665/31dee0d0a-5c85-4c3c-b68e-407930ebf4f0) | Asteraceae | *Silphium* | *perfoliatum* var. *connatum* | MF349334 | MF349874 | MF348816 | MF349184 |
| AD5JS67 | [Van Neste 620](http://n2t.net/ark:/65665/3ab8f1b43-5185-45f0-95cf-a3afc77774a5) | Asteraceae | *Solidago* | *juncea* | MF349729 | MF349832 | MF348438 | MF348908 |
| AD7LH97 | [Van Neste 792](http://n2t.net/ark:/65665/3dc55e649-7683-4185-92a7-593f2392b70a) | Asteraceae | *Solidago* | *odora* | MF349631 | MF350106 | MF348540 |  |
| AD3HI37 | [Van Neste 231](http://n2t.net/ark:/65665/307ec4b7d-3922-4790-9330-08df70273a06) | Asteraceae | *Spilanthes* | *oleracea* | MF349519 | MF350268 | MF348657 | MF349060 |
| AD0EG21 | [Van Neste 486](http://n2t.net/ark:/65665/3ecb3c832-45a2-469a-843a-a9cdc30c0d5a) | Asteraceae | *Stokesia* | *laevis* | MF349777 | MF350203 | MF348384 | MF348877 |
| AD3HK91 | [Van Neste 487](http://n2t.net/ark:/65665/372e47967-3b45-41f1-97df-99be4acaf5d5) | Asteraceae | *Stokesia* | *laevis* | MF349603 | MF350084 | MF348569 | MF348987 |
| AD7LH85 | [Van Neste 780](http://n2t.net/ark:/65665/317376435-6a33-4d30-b4fe-a8e079ef44a8) | Asteraceae | *Symphyotrichum* | *novae-angliae* | MF349639 | MF350111 | MF348530 | MF348966 |
| AD7LN78 | [Van Neste 711](http://n2t.net/ark:/65665/3914a3250-0a07-4330-bca8-43209ed07e19) | Asteraceae | *Vernonia* | *angustifolia* | MF349663 | MF350130 | MF348505 | MF348950 |
| AD3HL04 | [Van Neste 500](http://n2t.net/ark:/65665/3f4202a26-7b08-4a9c-80fb-802fa868b827) | Asteraceae | *Vernonia* | *glauca* | MF349558 | MF350052 | MF348619 | MF349028 |
| AD3HK07 | [Van Neste 401](http://n2t.net/ark:/65665/3c5c9c815-f278-4f85-a461-9eb18aca1275) | Asteraceae | *Vernonia* | *noveboracensis* | MF349587 | MF350073 | MF348584 | MF348999 |
| AD0EG88 | [Van Neste 525](http://n2t.net/ark:/65665/360b046ef-0b06-4848-be8f-f025fb1046a9) | Athyriaceae | *Athyrium* | *filix-femina* | MF349772 |  | MF348395 |  |
| AC9DD04 | [Van Neste 203](http://n2t.net/ark:/65665/37c677737-c19c-4128-b7df-057ff509094d) | Begoniaceae | *Begonia* | *nelumbiifolia* | MF349775 | MF350201 | MF348388 | MF348878 |
| AG2KK99 | [Ingram 7](http://n2t.net/ark:/65665/3f4c5c61c-ba50-41c8-bb87-aedbfc95c428) | Berberidaceae | *Caulophyllum* | *thallictroides* | MF349344 |  | MF348811 |  |
| AD7LL47 | [Sedaghatpour 2016-35](http://n2t.net/ark:/65665/30294cbc7-bff7-4feb-b0f6-a1afd1b6986e) | Berberidaceae | *Epimedium* | spp. | MF349221 |  | MF348762 | MF349132 |
| AG2KK93 | [Ingram 4](http://n2t.net/ark:/65665/3e25e6ae4-a372-4ff3-813f-cc20985b7347) | Berberidaceae | *Jeffersonia* | *diphylla* | MF349364 | MF349896 |  | MF349164 |
| AD7LM65 | [Sedaghatpour 2016-52](http://n2t.net/ark:/65665/330ba695d-6b88-448a-b8e0-ac76781d801e) | Berberidaceae | *Mahonia* | *bealei* | MF349431 | MF349957 |  | MF349117 |
| AD7LM49 | [Gostel 182](http://n2t.net/ark:/65665/3e8d7ee33-0152-4539-997a-99ce5e6dd143) | Berberidaceae | *Nandina* | *domestica* var. *leucocarpa* | MF349423 |  | MF348748 |  |
| AD3HK82 | [Van Neste 478](http://n2t.net/ark:/65665/361fd7096-9180-49d4-9202-a24fc2e35646) | Berberidaceae | *Podophyllum* | *peltatum* | MF349551 | MF350044 | MF348628 | MF349038 |
| AD3HJ03 | [Van Neste 297](http://n2t.net/ark:/65665/3c1eb36b0-2cdf-4f6a-89cc-4ee5b487e29e) | Betulaceae | *Alnus* | *hirsuta* | MF349535 | MF350033 | MF348642 | MF349049 |
| AD7LI00 | [Van Neste 795](http://n2t.net/ark:/65665/3ac68baab-abf9-46c6-8364-c5ae843ccb0a) | Betulaceae | *Alnus* | *incana* | MF349622 | MF350275 | MF348550 |  |
| AD7LM16 | [Faulconer 30](http://n2t.net/ark:/65665/31af46a7b-e73c-43a7-88c2-144001ad577c) | Betulaceae | *Betula* | *nigra* | MF349398 | MF349925 | MF348775 | MF349142 |
| AD7LH33 | [Van Neste 756](http://n2t.net/ark:/65665/333e5bb3f-f18a-45ba-b9c2-1c1a9881bf0b) | Betulaceae | *Carpinus* | *caroliniana* | MF349624 | MF350098 | MF348548 | MF348977 |
| AD0EE23 | [Van Neste 312](http://n2t.net/ark:/65665/33d9d070b-2986-4431-896e-176511752777) | Betulaceae | *Carpinus* | *turczaninovii* | MF349810 | MF350231 | MF348340 |  |
| AD3HJ17 | [Van Neste 312](http://n2t.net/ark:/65665/33d9d070b-2986-4431-896e-176511752777) | Betulaceae | *Carpinus* | *turczaninovii* | MF349526 | MF350028 | MF348652 | MF349055 |
| AD7LN05 | [Faulconer 50](http://n2t.net/ark:/65665/3e82fda98-c676-4b12-a7e7-655602eb97ae) | Betulaceae | *Corylus* | *avellena* | MF349393 | MF349920 | MF348778 | MF349146 |
| AG2KK95 | [Ingram 5](http://n2t.net/ark:/65665/3ac3e1e15-9ca3-402f-b830-e83e85a8e281) | Betulaceae | *Ostrya* | *virginiana* | MF349354 | MF349889 | MF348804 | MF349172 |
| AD0EF07 | [Van Neste 384](http://n2t.net/ark:/65665/3547e8a1e-3a88-4d70-9703-a090eb0393ca) | Bignoniaceae | *Campsis* | *radicans* | MF349767 | MF350194 | MF348401 |  |
| AG2KL15 | [Sedaghatpour 2016-61](http://n2t.net/ark:/65665/3e87cf78e-f176-49a1-992a-f5f04c19b712) | Bignoniaceae | *Podranea* | *ricasoliana* | MF349353 | MF349888 | MF348805 |  |
| AB6QQ17 | [Van Neste 117](http://n2t.net/ark:/65665/37eb2ce50-438b-41c2-9ed8-7d6a7b5b5be1) | Blechnaceae | *Blechnum* | *brasiliense* | MF349760 |  | MF348407 |  |
| AD5JS60 | [Van Neste 613](http://n2t.net/ark:/65665/3e92a3b66-f4de-47a6-bcec-712779b3daec) | Blechnaceae | *Woodwardia* | *areolata* | MF349280 |  | MF348478 |  |
| AD7LL63 | [Sedaghatpour 2016-43](http://n2t.net/ark:/65665/33307bc99-bd1d-414b-8a90-89d0ed6042d6) | Boraginaceae | *Brunnera* | *macrophylla* | MF349409 | MF349935 | MF348763 | MF349133 |
| AD7LM41 | [Gostel 178](http://n2t.net/ark:/65665/3f6f5d5ca-d87b-4bcb-8aed-55328835f796) | Boraginaceae | *Mertensia* | *virginiana* | MF349458 | MF349986 | MF348710 | MF349093 |
| AG2KK05 | [Gostel 196](http://n2t.net/ark:/65665/36ff97870-bcbb-4faa-84c2-caa8e53ce994) | Boraginaceae | *Myosotis* | spp. | MF349240 | MF349867 | MF348824 | MF349191 |
| AD7LL57 | [Sedaghatpour 2016-40](http://n2t.net/ark:/65665/3db0affc1-ecc2-46ff-a925-8ab795d14a5d) | Boraginaceae | *Pulmonaria* | *longifolia* |  | MF350251 | MF348737 | MF349113 |
| AG2KM47 | [Marcelli 28](http://n2t.net/ark:/65665/34c3c7c07-a043-4fd5-99b8-ce74479371fa) | Brassicaceae | *Erysimum* | *cheiri* | MF349308 | MF349856 | MF348842 | MF349206 |
| AD7LL65 | [Sedaghatpour 2016-44](http://n2t.net/ark:/65665/35f66d80a-e55e-439e-8673-d3ee78b17f5e) | Brassicaceae | *Iberis* | *sempervirens* | MF349399 | MF349926 | MF348774 | MF349141 |
| AD0EG20 | [Kelloff 1582](http://n2t.net/ark:/65665/3e9d07d82-03eb-4c17-83e8-45cf6fd1238b) | Brassicaceae | *Lepidium* | *virginicum* | MF349208 |  | MF348347 | MF348853 |
| AD3HL65 | [Kelloff 1582](http://n2t.net/ark:/65665/3e9d07d82-03eb-4c17-83e8-45cf6fd1238b) | Brassicaceae | *Lepidium* | *virginicum* | MF349581 |  | MF348590 | MF349004 |
| AD0ED77 | [Van Neste 270](http://n2t.net/ark:/65665/3f1c4d667-e270-48ee-840a-f54864f049fc) | Bromeliaceae | *Pitcairnia* | *sanguinea* | MF349295 |  |  |  |
| AG2KK37 | [Gostel 212](http://n2t.net/ark:/65665/3b1e1076e-4762-4288-85ed-658543f63e44) | Solanaceae | *Brugmansia* | *suaveolens* | MF349357 | MF349891 |  |  |
| AB6QQ32 | [Van Neste 133](http://n2t.net/ark:/65665/3bb84133e-d483-4345-8ed5-938528bab37e) | Buxaceae | *Buxus* | *microphylla* | MF349741 | MF350175 | MF348425 |  |
| AD7LN60 | [Van Neste 729](http://n2t.net/ark:/65665/3c1d58815-aba8-4007-a87a-edfed013e32e) | Buxaceae | *Pachysandra* | *procumbens* | MF349617 | MF350093 | MF348555 |  |
| AD7LH81 | [Van Neste 776](http://n2t.net/ark:/65665/37aa82e28-2542-45ef-930e-63c44eadbffa) | Buxaceae | *Sarcococca* | *hookeriana* | MF349659 | MF350127 | MF348510 |  |
| AD7LN23 | [Faulconer 59](http://n2t.net/ark:/65665/37c90aa08-cb26-4e84-a6b7-9bcb48e64d67) | Cactaceae | *Disocactus* | spp. | MF349237 | MF349982 | MF348712 |  |
| AD3HI75 | [Van Neste 269](http://n2t.net/ark:/65665/360d88cf7-3db7-4c2e-a7a9-cd0d33341446) | Cactaceae | *Lepismium* | *cruciforme* | MF349468 | MF349992 |  |  |
| AD0ED72 | [Van Neste 265](http://n2t.net/ark:/65665/334260235-b568-4653-856c-16a97a8ff538) | Cactaceae | *Rhipsalis* | *baccifera* | MF349781 | MF350207 | MF348378 |  |
| AD3HI72 | [Van Neste 266](http://n2t.net/ark:/65665/3c8408a4b-0955-442f-a9b6-6b59c917b835) | Cactaceae | *Rhipsalis* | *neves-armondii* | MF349484 | MF350002 | MF348685 |  |
| AG2KL77 | [Ingram 17](http://n2t.net/ark:/65665/3f7cf8a39-41ed-483b-b7c3-884459d9a82d) | Calycanthaceae | *Calycanthus* | *floridus* | MF349320 | MF349861 | MF348831 | MF349198 |
| AD7LK10 | [Gostel 169](http://n2t.net/ark:/65665/3ecabb5b9-d952-4d39-8484-a6fb376d95af) | Campanulaceae | *Adenophora* | *liliifolioides* | MF349244 | MF349852 | MF348753 | MF349126 |
| AD5JS48 | [Van Neste 601](http://n2t.net/ark:/65665/35405175f-8a1c-422b-92a1-8575baf01c41) | Campanulaceae | *Campanula* | *aparinoides* | MF349721 | MF350162 | MF348447 | MF348913 |
| AC9DD27 | [Van Neste 226](http://n2t.net/ark:/65665/38887ccbd-0a0d-4f8b-8015-c346702908a5) | Campanulaceae | *Codonopsis* | *pilosula* | MF349299 | MF350215 | MF348368 | MF348869 |
| AD3HI32 | [Van Neste 226](http://n2t.net/ark:/65665/38887ccbd-0a0d-4f8b-8015-c346702908a5) | Campanulaceae | *Codonopsis* | *pilosula* | MF349531 | MF350030 | MF348648 | MF349052 |
| AD3HL45 | [Van Neste 541](http://n2t.net/ark:/65665/38b7dd03b-4b0b-450d-b0a1-fb9a966e0616) | Campanulaceae | *Lobelia* | *cardinalis* | MF349638 | MF350276 | MF348533 |  |
| AD3HJ24 | [Van Neste 318](http://n2t.net/ark:/65665/347043b75-7878-4937-b910-f449ca100d4a) | Campanulaceae | *Platycodon* | *grandiflorus* | MF349504 | MF350013 |  | MF349068 |
| AD7LN82 | [Van Neste 707](http://n2t.net/ark:/65665/334fc8ea0-0bdb-471a-b69b-8bcc612949e8) | Canellaceae | *Warburgia* | *salutaris* | MF349682 | MF350144 | MF348484 | MF348939 |
| AD0EF04 | [Van Neste 382](http://n2t.net/ark:/65665/3f7c5c86a-7056-41fb-b13f-bab5a2dc076f) | Cannabaceae | *Celtis* | *laevigata* | MF349807 | MF350229 | MF348343 |  |
| AD3HL41 | [Van Neste 537](http://n2t.net/ark:/65665/3f101a41b-bd08-41b0-b55a-fa66e5934fb9) | Caprifoliaceae | *Abelia* | *grandiflora* | MF349646 | MF350118 | MF348523 |  |
| AD7LN61 | [Van Neste 728](http://n2t.net/ark:/65665/3741cce1e-c9a0-4670-95fa-f1f87f0dfa3f) | Caprifoliaceae | *Diervilla* | *rivularis* | MF349626 | MF350100 | MF348546 |  |
| AG2KK03 | [Gostel 195](http://n2t.net/ark:/65665/3e213d4f4-0c48-45ce-829b-1ba9984efc46) | Caprifoliaceae | *Knautia* | *macedonica* | MF349337 | MF350248 |  | MF349181 |
| AD7LH28 | [Van Neste 751](http://n2t.net/ark:/65665/3512f9853-df97-4b1b-80fa-9443cbb3cea0) | Caprifoliaceae | *Lonicera* | *maackii* | MF349660 |  | MF348508 |  |
| AD5JS69 | [Van Neste 622](http://n2t.net/ark:/65665/390cb4111-eb4e-456b-af55-5a315fe554c9) | Caprifoliaceae | *Sambucus* | *canadensis* | MF349707 | MF350157 | MF348460 | MF348920 |
| AD7LH30 | [Van Neste 753](http://n2t.net/ark:/65665/3625ff915-6a77-4ac7-aec0-eb7a0685c37c) | Caprifoliaceae | *Viburnum* | *dilatatum* | MF349276 |  | MF348519 |  |
| AD0EE90 | [Van Neste 368](http://n2t.net/ark:/65665/3abd2ee97-c5b5-4daa-811b-0d3eef394945) | Caprifoliaceae | *Viburnum* | *prunifolium* | MF349293 | MF350199 | MF348391 |  |
| AD3HJ26 | [Van Neste 320](http://n2t.net/ark:/65665/31efe0297-d556-4348-b9cd-7d7fbf361168) | Caprifoliaceae | *Weigela* | *florida* | MF349492 | MF350004 | MF348681 |  |
| AD7LM89 | [Faulconer 42](http://n2t.net/ark:/65665/383e61cf7-a762-4a13-aa00-8bf1861a5b33) | Caryophyllaceae | *Dianthus* | *gratianopolitanus* | MF349394 | MF349921 |  | MF349145 |
| AG2KM11 | [Marcelli 19](http://n2t.net/ark:/65665/3907eee55-a7cb-4db9-8ecb-35c473ea946e) | Caryophyllaceae | *Lychnis* | *coronaria* | MF349319 |  | MF348832 | MF349199 |
| AD5JP73 | [Kelloff 1590](http://n2t.net/ark:/65665/307a75bd0-7d82-4ef9-96d8-d42d9a0958d0) | Caryophyllaceae | *Silene* | *latifolia* | MF349814 |  | MF348335 | MF348849 |
| AD7LI86 | [Kelloff 1648](http://n2t.net/ark:/65665/39d0c9005-d13d-4392-a126-6ea0dcf5f9f7) | Celastraceae | *Celastrus* | *orbiculatus* | MF349554 | MF350048 | MF348623 | MF349033 |
| AD3HL34 | [Van Neste 530](http://n2t.net/ark:/65665/349b2d5cd-e87b-4a79-95a2-39dd9fa29f1d) | Celastraceae | *Euonymus* | *americanus* | MF349674 | MF350139 |  |  |
| AD5JP82 | [Kelloff 1599](http://n2t.net/ark:/65665/3aad05af3-be9b-4099-b7b9-31384a63fdff) | Cistaceae | *Helianthemum* | *canadense* | MF349764 | MF349826 |  |  |
| AD0EF41 | [Van Neste 417](http://n2t.net/ark:/65665/39cde9d2b-1287-467f-8e64-6ff2e7baa1b3) | Clethraceae | *Clethra* | *alnifolia* | MF349291 | MF350258 | MF348393 |  |
| AD5JP62 | [Van Neste 564](http://n2t.net/ark:/65665/342528a13-9666-4d81-b041-e8fa43643c20) | Clusiaceae | *Hypericum* | *canadense* | MF349752 |  | MF348415 | MF348891 |
| AD5JS58 | [Van Neste 611](http://n2t.net/ark:/65665/3615576c9-452f-4df6-a7f5-0ea050212103) | Clusiaceae | *Hypericum* | *hypericoides* | MF349708 |  | MF348459 | MF348919 |
| AD5JR00 | [Van Neste 586](http://n2t.net/ark:/65665/3484f62e9-e8b7-49b3-a8d7-4f442d1eca37) | Commelinaceae | *Aneilema* | *gillettii* | MF349691 | MF350148 |  |  |
| AD5JQ96 | [Van Neste 582](http://n2t.net/ark:/65665/301bfe01c-ab39-4b94-be4f-fe7fb7b518d4) | Commelinaceae | *Coleotrype* | *natalensis* | MF349710 |  | MF348457 |  |
| AD3HJ95 | [Van Neste 389](http://n2t.net/ark:/65665/33d12cd59-7ee8-4a98-a6f9-a9676dffc719) | Commelinaceae | *Commelina* | *communis* | MF349606 |  | MF348565 |  |
| AD3HK79 | [Van Neste 475](http://n2t.net/ark:/65665/3e3cccba8-4985-49a6-b5e5-881bca67b006) | Commelinaceae | *Commelina* | *communis* | MF349575 |  | MF348598 | MF349016 |
| AB6QQ03 | [Van Neste 103](http://n2t.net/ark:/65665/302df52fd-abda-4781-8a80-d18eb85f8af3) | Commelinaceae | *Cyanotis* | *nyctitropa* | MF349761 | MF350190 | MF348406 |  |
| AB6QQ04 | [Van Neste 104](http://n2t.net/ark:/65665/32bb4b4de-5051-4f86-8d0d-1aa87a013ecf) | Commelinaceae | *Cyanotis* | *villosa* | MF349754 | MF349828 | MF348413 |  |
| AD3HI95 | [Van Neste 289](http://n2t.net/ark:/65665/3ed3e5480-63e6-4b87-a7a3-53e5742291a5) | Commelinaceae | *Dichorisandra* | *penduliflora* | MF349483 |  | MF348686 |  |
| AD3HI17 | [Van Neste 211](http://n2t.net/ark:/65665/3a79c4a4b-7af1-4f74-a5c7-6f7018a52fa0) | Commelinaceae | *Dichorisandra* | *thyrsiflora* | MF349487 |  |  |  |
| AD5JQ92 | [Van Neste 578](http://n2t.net/ark:/65665/39a547ca7-f4aa-43cb-bfec-0baeab4eef29) | Commelinaceae | *Gibasis* | *pellucida* |  | MF349831 | MF348427 |  |
| AG2KJ95 | [Gostel 191](http://n2t.net/ark:/65665/39ea0717e-6333-447f-84f6-6d479321bdd7) | Commelinaceae | *Tinantia* | *pringlei* | MF349369 | MF349900 | MF348793 |  |
| AD7LN13 | [Faulconer 54](http://n2t.net/ark:/65665/3fc598ea9-573f-47b5-a29d-2852e2c472e3) | Commelinaceae | *Tradescantia* | *ernestiana* | MF349428 |  | MF348743 |  |
| AG2KL65 | [Ingram 11](http://n2t.net/ark:/65665/3ebab4862-16ec-48b5-99ec-95f04a9277b4) | Compositae | *Achillea* | *millefolium* | MF349338 | MF349876 | MF348813 | MF349180 |
| AG2KK29 | [Gostel 208](http://n2t.net/ark:/65665/3ed543f1f-f03d-4542-9b13-75793917c468) | Compositae | *Dahlia* | spp. | MF349241 | MF349860 |  | MF349201 |
| AG2KJ93 | [Gostel 190](http://n2t.net/ark:/65665/39f0dd396-d24d-4858-882a-8d8a4dda63c0) | Compositae | *Emilia* | *javonica* | MF349379 | MF349910 | MF348787 | MF349157 |
| AG2KK17 | [Gostel 202](http://n2t.net/ark:/65665/32d396818-0d9a-4181-b875-7f2ffd7a0769) | Compositae | *Helenium* | *amarum* | MF349368 | MF349899 | MF348794 | MF349162 |
| AG2KJ97 | [Gostel 192](http://n2t.net/ark:/65665/3f1b13c36-55d1-4598-a0f0-e0484f3e739f) | Compositae | *Santolina* | *rosmarinifolia* | MF349359 | MF349892 | MF348801 | MF349169 |
| AG2KM05 | [Marcelli 16](http://n2t.net/ark:/65665/329bde478-4c3a-4611-b69c-bfacfd1b4f78) | Compositae | *Zinnia* | *elegans* | MF349340 | MF349878 | MF348812 | MF349178 |
| AD0EE82 | [Van Neste 359](http://n2t.net/ark:/65665/3bd028e3b-8e2d-4331-87b2-400ee9818f2e) | Convolvulaceae | *Calystegia* | *sepium* | MF349788 | MF350212 | MF348372 | MF348871 |
| AB6QQ26 | [Van Neste 127](http://n2t.net/ark:/65665/3d5f44872-189f-4d8d-98a9-7a3a9049db2f) | Cornaceae | *Aucuba* | *japonica* | MF349723 | MF350164 | MF348443 | MF348911 |
| AD7LN86 | [Van Neste 703](http://n2t.net/ark:/65665/3a2bffa87-76c8-4888-bd44-f0f78111bd94) | Cornaceae | *Camptotheca* | *acuminata* | MF349628 | MF350102 | MF348544 | MF348976 |
| AD3HJ08 | [Van Neste 302](http://n2t.net/ark:/65665/35e66f7c9-4db4-4e3a-a9be-23794a21053f) | Cornaceae | *Cornus* | *florida* | MF349515 | MF350021 | MF348660 |  |
| AC9DD67 | [Van Neste 539](http://n2t.net/ark:/65665/399d400c1-26ec-4f7d-85b1-ebadef830a16) | Cornaceae | *Cornus* | *mas* | MF349302 |  |  |  |
| AD5JT35 | [Van Neste 662](http://n2t.net/ark:/65665/3266c3e89-d7a6-4224-b53e-98ffb56b3d21) | Cornaceae | *Cornus* | *sanguinea* | MF349745 | MF349829 | MF348421 | MF348897 |
| AD5JP52 | [Van Neste 554](http://n2t.net/ark:/65665/34ce9d17b-ec15-4138-be4c-bb41f40f6dc5) | Cornaceae | *Cornus* | *sericea* | MF349701 | MF349838 | MF348466 |  |
| AD5JS85 | [Van Neste 675](http://n2t.net/ark:/65665/3e50bba5e-c05a-4503-b474-d495321d57c0) | Crassulaceae | *Adromischus* | *marianae* | MF349687 | MF349842 | MF348482 | MF348938 |
| AD5JS87 | [Van Neste 677](http://n2t.net/ark:/65665/30cab0ff8-7fef-44aa-a642-1f605d7f9d42) | Crassulaceae | *Crassula* | *perfoliata* | MF349744 |  | MF348422 | MF348898 |
| AD5JS89 | [Van Neste 679](http://n2t.net/ark:/65665/303db85ba-61ab-49a0-ae4c-ef88ace31a5c) | Crassulaceae | *Crassula* | *rupestris* | MF349725 |  | MF348441 |  |
| AD5JS90 | [Van Neste 680](http://n2t.net/ark:/65665/33aa78710-bea6-4579-b8e9-c28993b4251a) | Crassulaceae | *Echeveria* | *setosa* | MF349715 |  | MF348452 | MF348916 |
| AD5JS86 | [Van Neste 676](http://n2t.net/ark:/65665/34bf592f8-8aef-4f31-8467-eeb5ecebd0c4) | Crassulaceae | *Echeveria* | *shaviana* |  |  |  | MF348889 |
| AD5JS91 | [Van Neste 681](http://n2t.net/ark:/65665/314615111-fb58-44a8-b2ca-9fa536af56f7) | Crassulaceae | *Graptopetalum* | *pentandrum* | MF349703 |  | MF348464 | MF348924 |
| AD0EE70 | [Van Neste 353](http://n2t.net/ark:/65665/3ce6ec307-25be-4793-876e-42dd63f2b341) | Crassulaceae | *Phedimus* | *aizoon* | MF349798 |  | MF348358 | MF348861 |
| AD5JR12 | [Van Neste 353](http://n2t.net/ark:/65665/3ce6ec307-25be-4793-876e-42dd63f2b341) | Crassulaceae | *Phedimus* | *aizoon* | MF349251 |  | MF348645 | MF349050 |
| AD7LM95 | [Faulconer 45](http://n2t.net/ark:/65665/395bf5963-77d2-4e85-a0ec-6770a5e21097) | Cupressaceae | *Chamaecyparis* | *obtusa* | MF349437 |  | MF348731 | MF349108 |
| AD7LM32 | [Faulconer 38](http://n2t.net/ark:/65665/3abbc1039-af08-4eff-b0d6-330e5d49f004) | Cupressaceae | *Chamaecyparis* | *thyoides* | MF349415 |  | MF348755 | MF349127 |
| AD7LM59 | [Sedaghatpour 2016-49](http://n2t.net/ark:/65665/30da6e18e-130d-4316-8f7f-f6a0dbbdd29b) | Cupressaceae | *Cryptomeria* | *japonica* | MF349457 |  |  | MF349094 |
| AG2KM43 | [Sedaghatpour 2016-87](http://n2t.net/ark:/65665/3f3e1e6ce-dbd7-43e5-af95-5b622af98b74) | Cupressaceae | *Cupressus* | *arizonica* | MF349318 |  | MF348833 | MF349200 |
| AD7LM71 | [Sedaghatpour 2016-55](http://n2t.net/ark:/65665/361dd4d14-b1b8-47b0-8ba8-ddc9657b8323) | Cupressaceae | *Cunninghamia* | *lanceolata* | MF349405 |  | MF348767 | MF349136 |
| AG2KL93 | [Sedaghatpour 2016-74](http://n2t.net/ark:/65665/3b8a04155-0694-403a-8689-66226390733b) | Cycadaceae | *Cycas* | *circinalis* |  |  | MF348799 | MF349167 |
| AD5JQ56 | [Kelloff 1595](http://n2t.net/ark:/65665/32612a8ae-ca3b-4551-b361-db7207e9a3bc) | Cyperaceae | *Bulbostylis* | *ciliatifolia* | MF349590 |  |  | MF348997 |
| AD3HJ91 | [Van Neste 385](http://n2t.net/ark:/65665/3a9652056-4fc0-41ca-bc30-beb2e4526d9b) | Cyperaceae | *Carex* | *annectens* | MF349255 | MF350047 | MF348624 | MF349034 |
| AD7LH73 | [Van Neste 768](http://n2t.net/ark:/65665/3db8aefd5-e37c-4a29-84b6-f29561929ee4) | Cyperaceae | *Carex* | *glaucescens* | MF349616 |  |  |  |
| AD5JS62 | [Van Neste 615](http://n2t.net/ark:/65665/3baf4df15-42b4-4d14-a1a4-568b783457fc) | Cyperaceae | *Cyperus* | *echinatus* | MF349757 | MF350185 |  |  |
| AD0EE78 | [Kelloff 1585](http://n2t.net/ark:/65665/3f1f5befb-bf74-4271-a05b-731804fd2a96) | Cyperaceae | *Cyperus* | *lancastriensis* | MF349762 | MF350192 | MF348404 |  |
| AD7LI82 | [Kelloff 1644](http://n2t.net/ark:/65665/3f76c1d5f-e255-463f-834e-74ab03f6dbd4) | Cyperaceae | *Eleocharis* | *engelmannii* | MF349580 | MF350264 |  | MF349007 |
| AD3HJ87 | [Van Neste 381](http://n2t.net/ark:/65665/3f0929978-3efa-44b5-aff4-c3c7f41b7992) | Cyperaceae | *Kyllinga* | *gracillima* | MF349258 | MF350054 | MF348614 | MF349023 |
| AD5JS56 | [Van Neste 609](http://n2t.net/ark:/65665/339db0632-8066-46f7-8b4d-39bcc0e77e8f) | Cyperaceae | *Rhynchospora* | *capitellata* |  |  |  | MF348907 |
| AD3HK59 | [Van Neste 456](http://n2t.net/ark:/65665/3bdfa0803-3d5f-490f-b0c5-a198c5b67ee4) | Cyperaceae | *Schoenoplectus* | *validus* | MF349552 |  |  |  |
| AD5JS51 | [Van Neste 604](http://n2t.net/ark:/65665/369731f4f-a3f6-4462-8761-67aa64e7a663) | Cyperaceae | *Scirpus* | *expansus* | MF349699 | MF350259 |  | MF348929 |
| AD0EG57 | [Van Neste 516](http://n2t.net/ark:/65665/39c195884-2531-4cb6-9da9-596e423adc7a) | Cyrillaceae | *Cyrilla* | *racemiflora* | MF349806 | MF350228 | MF348344 |  |
| AD5JT31 | [Van Neste 658](http://n2t.net/ark:/65665/36c8f8cf2-f83d-4509-be1c-25c7fa46b605) | Dennstaedtiaceae | *Dennstaedtia* | *punctilobula* | MF349755 |  |  |  |
| AD5JP88 | [Kelloff 1605](http://n2t.net/ark:/65665/31600a5d8-41b4-4e02-a70d-cf02f6df97ba) | Dennstaedtiaceae | *Pteridium* | *aquilinum* | MF349296 |  | MF348387 |  |
| AD5JQ66 | [Kelloff 1605](http://n2t.net/ark:/65665/31600a5d8-41b4-4e02-a70d-cf02f6df97ba) | Dennstaedtiaceae | *Pteridium* | *aquilinum* | MF349253 |  | MF348630 |  |
| AD5JQ11 | [Kelloff 1628](http://n2t.net/ark:/65665/3e68bea4f-c7b3-4321-bf49-87e10a41f79b) | Dipsacaceae | *Dipsacus* | *fullonum* | MF349771 | MF350257 |  | MF348882 |
| AD7LM81 | [Vo 78](http://n2t.net/ark:/65665/3f6a8de8d-5fd9-413f-97be-7d30adcc81d8) | Dryopteraceae | *Onoclea* | *sensibilis* | MF349430 |  | MF348741 |  |
| AG2KK13 | [Gostel 200](http://n2t.net/ark:/65665/31b7e8ebe-8049-4bab-855c-9e2b5fd6b14d) | Dryopteridaceae | *Arachniodes* | *simplicior* | MF349378 |  |  |  |
| AD7LN09 | [Faulconer 52](http://n2t.net/ark:/65665/39d937a0b-6232-4e3f-9adc-1b1d88eaa1d7) | Dryopteridaceae | *Cyrtomium* | *fortunei* | MF349447 |  | MF348722 |  |
| AD3HK25 | [Van Neste 419](http://n2t.net/ark:/65665/35ca2aa0d-9872-4b45-a5d9-3ad19a81f095) | Dryopteridaceae | *Dryopteris* | *marginalis* | MF349568 |  |  |  |
| AD3HI54 | [Van Neste 248](http://n2t.net/ark:/65665/3c90f2fb0-ad32-4613-9955-35aeb0cd3df0) | Dryopteridaceae | *Elaphoglossum* | *paleaceum* | MF349517 |  | MF348658 |  |
| AD0EF39 | [Van Neste 415](http://n2t.net/ark:/65665/3507fbfa6-b5a2-47c7-b774-c38aa8784dec) | Dryopteridaceae | *Polystichum* | *acrostichoides* | MF349795 |  | MF348362 |  |
| AD7LH87 | [Van Neste 782](http://n2t.net/ark:/65665/3c7f2a61e-ecc0-4927-a77c-0ed58e66d722) | Dryopteridaceae | *Polystichum* | *acrostichoides* | MF349269 |  | MF348549 |  |
| AG2KJ83 | [Gostel 185](http://n2t.net/ark:/65665/3a4c4e68f-733d-4e9b-9a38-34f8205594ae) | Ebenaceae | *Diospyros* | *virginiana* | MF349402 | MF349929 |  |  |
| AD3HL24 | [Van Neste 520](http://n2t.net/ark:/65665/340448cd7-1f04-438f-bfc4-8d0ed0851677) | Ericaceae | *Agarista* | *populifolia* | MF349272 | MF350109 | MF348532 | MF348968 |
| AD7LM36 | [Faulconer 40](http://n2t.net/ark:/65665/36ce1bdb5-76b0-4d42-9cfb-8fcfeff37d38) | Ericaceae | *Chamaedaphne* | *calyculata* | MF349407 | MF349933 | MF348765 |  |
| AD3HK77 | [Van Neste 473](http://n2t.net/ark:/65665/3f9a60f18-00dd-4386-b14b-d62f76be40e2) | Ericaceae | *Chimaphila* | *maculata* | MF349584 | MF350070 | MF348587 | MF349002 |
| AD5JS64 | [Van Neste 617](http://n2t.net/ark:/65665/32d4e884b-586c-4ed2-a223-b78c13858a5c) | Ericaceae | *Kalmia* | *latifolia* | MF349748 | MF350180 | MF348418 | MF348894 |
| AD5JS53 | [Van Neste 606](http://n2t.net/ark:/65665/3a116a852-d23b-4c70-9259-1fd05e2e4b9b) | Ericaceae | *Leucothoe* | *racemosa* | MF349758 | MF350186 | MF348410 | MF348887 |
| AD7LI39 | [Van Neste 814](http://n2t.net/ark:/65665/3f96a0183-0d61-48a2-806b-f3ec8f3d6be4) | Ericaceae | *Macleania* | *insignis* | MF349277 |  | MF348502 | MF348949 |
| AB6QQ23 | [Van Neste 124](http://n2t.net/ark:/65665/3f2521531-80ea-4d2c-9404-33873f31e478) | Ericaceae | *Macleania* | *smithiana* |  |  |  | MF348905 |
| AD0EG47 | [Kelloff 1576](http://n2t.net/ark:/65665/37c77415b-a04a-4511-b133-e5d0d0963f80) | Ericaceae | *Monotropa* | *uniflora* |  |  |  | MF348876 |
| AD3HL18 | [Van Neste 514](http://n2t.net/ark:/65665/3f31c2c4e-e572-41c0-92f7-e74cff9f8a06) | Ericaceae | *Oxydendrum* | *arboreum* | MF349539 |  | MF348639 | MF349046 |
| AD7LM57 | [Sedaghatpour 2016-48](http://n2t.net/ark:/65665/379e39daf-353b-43cd-8eaa-ea519574173c) | Ericaceae | *Pieris* | *japonica* | MF349396 | MF349923 | MF348776 | MF349144 |
| AD3HL22 | [Van Neste 518](http://n2t.net/ark:/65665/3564d07f8-e287-4889-a313-6ba9cb35341d) | Ericaceae | *Vaccinium* | *macrocarpon* | MF349647 |  | MF348522 | MF348962 |
| AG2KK65 | [Marcelli 4](http://n2t.net/ark:/65665/30ae60dac-86dd-4216-a0d4-3c4b74df5642) | Ericaceae | *Zenobia* | *pulverulenta* | MF349315 | MF349859 | MF348835 | MF349202 |
| AG2KL19 | [Sedaghatpour 2016-63](http://n2t.net/ark:/65665/3ed361064-1e93-499c-9d65-c32b340f1b2d) | Euphorbiaceae | *Codieaum* | *variegatum* | MF349331 | MF350246 | MF348818 | MF349187 |
| AD0EE80 | [Kelloff 1587](http://n2t.net/ark:/65665/3abe50e64-b6cd-424f-af47-07aace242c3d) | Fabaceae | *Baptisia* | *leucantha* | MF349763 | MF350191 | MF348405 | MF348845 |
| AG2KK27 | [Gostel 207](http://n2t.net/ark:/65665/3788087dd-9cb4-4a2a-8194-69f228c005bc) | Fabaceae | *Indigofera* | *kirilowii* | MF349326 | MF350245 |  | MF349192 |
| AG2KM27 | [Sedaghatpour 2016-79](http://n2t.net/ark:/65665/3ff7bf789-6ce9-43e1-a859-79d5c75cb615) | Fabaceae | *Wisteria* | *frutescens* | MF349360 | MF349893 | MF348800 | MF349168 |
| AD7LH15 | [Van Neste 738](http://n2t.net/ark:/65665/3584e91f6-d343-4cc5-9874-57272b58ee48) | Fagaceae | *Castanea* | spp. | MF349219 | MF350107 | MF348537 |  |
| AG2KL83 | [Ingram 20](http://n2t.net/ark:/65665/39d180205-98ea-49ef-831d-67fc9ad6b827) | Fagaceae | *Fagus* | *grandifolia* | MF349381 |  | MF348840 | MF349154 |
| AD0EF09 | [Van Neste 409](http://n2t.net/ark:/65665/301f2bd81-38ea-45cf-8cb5-10a42eec0ef5) | Fagaceae | *Quercus* | *acutissima* | MF349306 | MF350236 | MF348331 |  |
| AD3HK15 | [Van Neste 409](http://n2t.net/ark:/65665/301f2bd81-38ea-45cf-8cb5-10a42eec0ef5) | Fagaceae | *Quercus* | *acutissima* | MF349546 |  | MF348634 | MF349041 |
| AD7LH17 | [Van Neste 740](http://n2t.net/ark:/65665/3f4fac762-f0c3-47ce-b808-0b79b8e816f8) | Fagaceae | *Quercus* | *acutissima* | MF349625 | MF350099 | MF348547 |  |
| AD0EF71 | [Van Neste 452](http://n2t.net/ark:/65665/342ad42fa-11b2-4ec4-aa3d-36dc43bf37d9) | Gentianaceae | *Sabatia* | *kennedyana* | MF349794 | MF350219 | MF348363 | MF348864 |
| AD7LM18 | [Faulconer 31](http://n2t.net/ark:/65665/3bd7b8fc3-190c-4eef-87d8-367f7cb3c8e0) | Geraniaceae | *Geranium* | *sanguineum* | MF349459 |  | MF348709 | MF349092 |
| AC9DD29 | [Van Neste 228](http://n2t.net/ark:/65665/34cc397d1-c47a-4198-8a4b-454d4fba56c9) | Geraniaceae | *Pelargonium* | *ionidiflorum* | MF349305 | MF350266 | MF348338 |  |
| AD7LH43 | [Van Neste 767](http://n2t.net/ark:/65665/30ea1a84b-4ad4-4a10-978f-9be10d06e50b) | Ginkgoaceae | *Ginkgo* | *biloba* | MF349623 |  |  |  |
| AD0EE37 | [Van Neste 325](http://n2t.net/ark:/65665/398a11e68-c460-4dac-b2ca-14f98d795948) | Hamamelidaceae | *Corylopsis* | *glandulifera* | MF349789 | MF350213 | MF348370 |  |
| AD7LM22 | [Faulconer 33](http://n2t.net/ark:/65665/31d95c298-5acf-4c6f-ba2f-c5579034f4e5) | Hamamelidaceae | *Fothergilla* | *gardinii* | MF349441 | MF349967 | MF348729 | MF349104 |
| AD7LM20 | [Faulconer 32](http://n2t.net/ark:/65665/3356f8ca0-4a2e-4a40-9f33-7d601e90ab9a) | Hamamelidaceae | *Fothergilla* | *gardinii* x *intermedia* | MF349390 | MF349977 | MF348717 | MF349100 |
| AD7LM47 | [Gostel 181](http://n2t.net/ark:/65665/348f3671b-c43a-4582-b5d7-49687258a7b9) | Hamamelidaceae | *Parrotia* | *persica* | MF349432 | MF349958 | MF348740 | MF349116 |
| AC9DD03 | [Van Neste 202](http://n2t.net/ark:/65665/3139f90e0-4603-4e93-a3e5-af059711bb76) | Heliconiaceae | *Heliconia* | *psittacorum* | MF349820 | MF350241 | MF348326 |  |
| AD0EF40 | [Van Neste 416](http://n2t.net/ark:/65665/3e60e926d-a43b-42ac-b0a7-74230b9c8a52) | Hippocastanaceae | *Aesculus* | *pavia* | MF349297 | MF350204 | MF348383 |  |
| AD0EE28 | [Van Neste 316](http://n2t.net/ark:/65665/329ff13a6-a37d-46ef-b698-ff256dc6cd19) | Hydrangeaceae | *Hydrangea* | *paniculata* | MF349811 | MF350232 | MF348339 | MF348851 |
| AD7LM53 | [Sedaghatpour 2016-46](http://n2t.net/ark:/65665/338787354-d943-487c-b8b2-fbb705ac9fad) | Hydrangeaceae | *Schizophragma* | *hydrangeoides* | MF349414 | MF349942 | MF348756 |  |
| AG2KK83 | [Marcelli 13](http://n2t.net/ark:/65665/3c36167d1-f180-4da0-a833-d4dc1ad87630) | Hypericaceae | *Hypericum* | *frondosum* | MF349333 |  | MF348817 | MF349185 |
| AB6QQ13 | [Van Neste 113](http://n2t.net/ark:/65665/3e1c0b8c8-658b-4387-994d-d84eba035f09) | Hypnaceae | *Hypnum* | *curvifolium* | MF349284 |  | MF348465 | MF348925 |
| AD7LL55 | [Sedaghatpour 2016-39](http://n2t.net/ark:/65665/3554e4dea-36a3-47a5-92b4-f66cc591733a) | Iridaceae | *Crocus* | *sativus* | MF349443 | MF349969 | MF348727 | MF349103 |
| AD0EE84 | [Van Neste 361](http://n2t.net/ark:/65665/3749147aa-67c7-47cb-b96f-644aa97983b2) | Iridaceae | *Iris* | *pseudacorus* | MF349817 | MF350237 | MF348330 |  |
| AG2KL79 | [Ingram 18](http://n2t.net/ark:/65665/39ea1fe65-6273-4051-8cbc-c1934506cba3) | Iridaceae | *Sisyrinchium* | spp. | MF349243 | MF349868 | MF348823 | MF349190 |
| AD7LM87 | [Vo 81](http://n2t.net/ark:/65665/38479de9f-6139-473b-8d58-c04b8f749ef1) | Juglandaceae | *Carya* | *glabra* | MF349404 | MF349931 | MF348768 | MF349137 |
| AD0EE08 | [Van Neste 298](http://n2t.net/ark:/65665/3726693cb-2f57-44e8-8248-f6bc15a32690) | Juglandaceae | *Platycarya* | *strobilacea* | MF349303 | MF349824 | MF348350 |  |
| AD0EF85 | [Van Neste 456](http://n2t.net/ark:/65665/3bdfa0803-3d5f-490f-b0c5-a198c5b67ee4) | Juncaceae | *Schoenoplectus* | *validus* | MF349233 | MF350286 | MF348345 |  |
| AD7LH90 | [Van Neste 785](http://n2t.net/ark:/65665/36102da1f-fc5c-4bf1-9e87-6bfa8b4ade54) | Lamiaceae | *Agastache* | *foeniculum* | MF349615 |  | MF348557 |  |
| AD7LN01 | [Faulconer 48](http://n2t.net/ark:/65665/306fc9333-94e4-4001-b9ab-4f7a844d100d) | Lamiaceae | *Ajuga* | *reptans* | MF349412 | MF349940 |  | MF349129 |
| AD3HK97 | [Van Neste 493](http://n2t.net/ark:/65665/3900c354f-968e-44fc-8812-eff3a8bf0c60) | Lamiaceae | *Callicarpa* | *americana* | MF349583 | MF350069 | MF348588 |  |
| AD5JP68 | [Van Neste 570](http://n2t.net/ark:/65665/38257bd91-0ab4-4149-8cd5-71b999f081a0) | Lamiaceae | *Cornutia* | *grandifolia* | MF349722 | MF349833 | MF348445 | MF348912 |
| AG2KM23 | [Marcelli 25](http://n2t.net/ark:/65665/3bafeb9f9-53be-4d65-bbfe-325548cc88c6) | Lamiaceae | *Hyssopus* | *officianlis* | MF349371 | MF349902 |  |  |
| AG2KJ89 | [Gostel 188](http://n2t.net/ark:/65665/3e8351987-19ae-44f9-b666-74d85c1e07d4) | Lamiaceae | *Lavandula* | spp. | MF349239 | MF350255 |  |  |
| AD7LI85 | [Kelloff 1647](http://n2t.net/ark:/65665/37c1c7864-ee4d-402a-bccb-0c267523a096) | Lamiaceae | *Lycopus* | *virginicus* | MF349562 | MF350270 | MF348613 | MF349022 |
| AG2KL03 | [Ingram 9](http://n2t.net/ark:/65665/3269c67e5-cc96-470d-9a07-997e7a243418) | Lamiaceae | *Meehania* | *cordata* | MF349332 | MF349873 |  | MF349186 |
| AD0EF78 | [Kelloff 1569](http://n2t.net/ark:/65665/353a2ce5c-5bb1-41b1-8b41-2a549a4efacd) | Lamiaceae | *Monarda* | *fistulosa* | MF349784 |  |  |  |
| AD3HL52 | [Kelloff 1569](http://n2t.net/ark:/65665/353a2ce5c-5bb1-41b1-8b41-2a549a4efacd) | Lamiaceae | *Monarda* | *fistulosa* | MF349566 | MF350057 | MF348609 | MF349018 |
| AG2KJ85 | [Gostel 186](http://n2t.net/ark:/65665/35683b3bd-d3d9-4237-ab5a-d91c564031d6) | Lamiaceae | *Nepeta* | spp. | MF349238 | MF349917 | MF348781 |  |
| AG2KK08 | [Gostel 197](http://n2t.net/ark:/65665/31a17056f-a9c9-48bb-8a33-ed5518d425ca) | Lamiaceae | *Nepeta* | spp. | MF349317 | MF350244 | MF348834 |  |
| AG2KL55 | [Vo 91](http://n2t.net/ark:/65665/3902e2b62-16b3-4397-b722-9e1bbb6d9861) | Lamiaceae | *Ocimum* | *basilicum* | MF349330 | MF349872 | MF348819 | MF349188 |
| AD5JT16 | [Van Neste 643](http://n2t.net/ark:/65665/3dbc55a27-d489-4510-b56f-843ff1bd4aae) | Lamiaceae | *Origanum* | *laevigatum* | MF349689 | MF350147 | MF348480 | MF348937 |
| AD5JP69 | [Van Neste 571](http://n2t.net/ark:/65665/372341ae7-5637-495a-83d8-bb30c66dfcfa) | Lamiaceae | *Petraeovitex* | *bambusetorum* | MF349711 | MF349837 | MF348456 |  |
| AD7LN67 | [Van Neste 722](http://n2t.net/ark:/65665/373cf8b3a-f594-4930-bad0-1102d5680841) | Lamiaceae | *Pycnanthemum* | *albescens* |  | MF350277 | MF348485 |  |
| AD7LL61 | [Sedaghatpour 2016-42](http://n2t.net/ark:/65665/300dc6e31-c4cd-4e1a-9172-66f2a835eba5) | Lamiaceae | *Rosmarinus* | *officinalis* | MF349416 | MF349943 | MF348754 |  |
| AD3HJ40 | [Van Neste 334](http://n2t.net/ark:/65665/3697a4d64-865d-428b-8fdd-a7d5d769f507) | Lamiaceae | *Salvia* | *miltiorhiza* | MF349486 |  | MF348684 |  |
| AG2KM17 | [Marcelli 22](http://n2t.net/ark:/65665/35de2ae54-099d-449e-8f8a-6652002855e9) | Lamiaceae | *Satureja* | *montana* | MF349309 | MF350242 | MF348841 | MF349205 |
| AD5JS76 | [Van Neste 629](http://n2t.net/ark:/65665/3b3ff6988-62ec-47ee-ad6c-0b16dc169d97) | Lamiaceae | *Scutellaria* | *elliptica* | MF349737 | MF350174 | MF348430 | MF348902 |
| AD3HK66 | [Van Neste 462](http://n2t.net/ark:/65665/38467c47d-ed27-45be-9f4a-7be786749804) | Lamiaceae | *Teucrium* | *chamaedrys* | MF349585 | MF350071 | MF348586 | MF349001 |
| AD5JT17 | [Van Neste 644](http://n2t.net/ark:/65665/3387a35cb-175a-4eca-9b3e-7d20c0be7f20) | Lamiaceae | *Thymus* | *serpyllum* | MF349756 | MF350183 | MF348412 | MF348888 |
| AD3HJ30 | [Van Neste 324](http://n2t.net/ark:/65665/3a18427e2-b34d-49ed-b009-eaadad1d57d4) | Lamiaceae | *Vitex* | *negundo* | MF349534 | MF350269 | MF348643 |  |
| AD7LL53 | [Sedaghatpour 2016-38](http://n2t.net/ark:/65665/3371590b6-bb11-4db5-9077-3ca8373e43a8) | Lauraceae | *Persea* | *borbonia* | MF349452 | MF349978 | MF348715 |  |
| AD7LM77 | [Vo 76](http://n2t.net/ark:/65665/320d41afa-1e32-43a9-9159-68000716cd88) | Lauraceae | *Sassafras* | *albidum* | MF349449 | MF349974 | MF348720 |  |
| AD3HK56 | [Van Neste 453](http://n2t.net/ark:/65665/38d8ea1c0-5706-4c14-9841-ed82dbd939e0) | Lemnaceae | *Spirodela* | *oligorrhiza* | MF349577 | MF350065 | MF348596 |  |
| AD3HK57 | [Van Neste 454](http://n2t.net/ark:/65665/3c4dde6a7-24c5-4b68-8951-f89c271d8824) | Lemnaceae | *Wolffia* | spp. | MF349213 | MF350060 | MF348607 |  |
| AD5JP64 | [Van Neste 566](http://n2t.net/ark:/65665/3ad641516-1778-4bfe-8bee-2b6fc83448fa) | Lentibulariaceae | *Pinguicula* | *primuliflora* | MF349742 | MF350176 | MF348424 | MF348899 |
| AD7LH91 | [Van Neste 786](http://n2t.net/ark:/65665/3b49ebd27-2f27-4c32-862c-8af62e329b55) | Liliaceae | *Allium* | *tuberosum* | MF349677 | MF350142 | MF348489 | MF348942 |
| AD3HL96 | [Van Neste 450](http://n2t.net/ark:/65665/396694405-44cf-4cd5-8c40-1359caf23d6e) | Liliaceae | *Aloe* | *rauhii* | MF349595 | MF350077 | MF348577 | MF348993 |
| AD7LM99 | [Faulconer 47](http://n2t.net/ark:/65665/3872195b7-672c-4fd6-bd11-0f467a79773f) | Liliaceae | *Bellevalia* | *pycnantha* | MF349420 | MF349948 | MF348750 | MF349123 |
| AD7LJ09 | [Gostel 158](http://n2t.net/ark:/65665/3235bacbe-6e1a-410f-9f33-511d1eb0de6b) | Liliaceae | *Bessera* | *elegans* | MF349417 | MF349944 |  | MF349125 |
| AD7LN58 | [Van Neste 733](http://n2t.net/ark:/65665/3e0088bfe-97a0-4778-9e5a-2d4a2cfc260e) | Liliaceae | *Danae* | *racemosa* | MF349670 | MF350136 | MF348496 |  |
| AD5JR09 | [Van Neste 595](http://n2t.net/ark:/65665/342b05503-bded-4c35-8b9a-5fc734968cbb) | Liliaceae | *Dianella* | *ensifolia* | MF349750 | MF350182 | MF348416 | MF348893 |
| AD7LM97 | [Faulconer 46](http://n2t.net/ark:/65665/32b3d4f8c-6a6d-4145-8d09-147941ef69a6) | Liliaceae | *Disporum* | *cantoniense* | MF349429 | MF350249 | MF348742 |  |
| AD5JS95 | [Van Neste 685](http://n2t.net/ark:/65665/32cc4e830-a417-450c-9382-3dcef13d7a35) | Liliaceae | *Drimiopsis* | *botryoides* |  |  | MF348492 |  |
| AC9DD07 | [Van Neste 207](http://n2t.net/ark:/65665/3bcdcadec-e371-42a3-92e9-89b6c7c0da02) | Liliaceae | *Harperocallis* | *flava* | MF349800 | MF350222 | MF348357 | MF348859 |
| AD3HI13 | [Van Neste 207](http://n2t.net/ark:/65665/3bcdcadec-e371-42a3-92e9-89b6c7c0da02) | Liliaceae | *Harperocallis* | *flava* | MF349520 | MF350024 | MF348656 |  |
| AD5JS99 | [Van Neste 689](http://n2t.net/ark:/65665/3706398e3-d293-4477-a79e-aa8fa00cdc9d) | Liliaceae | *Haworthia* | *reinwardtii* | MF349637 |  |  |  |
| AD5JS98 | [Van Neste 688](http://n2t.net/ark:/65665/3bbc17715-4ccd-4bcb-9735-bb9d6a8f8d39) | Liliaceae | *Ledebouria* | *revoluta* | MF349645 | MF350117 | MF348524 |  |
| AD7LI83 | [Kelloff 1645](http://n2t.net/ark:/65665/38d82fe3c-6224-4ff6-b448-d94297c6014c) | Liliaceae | *Liriope* | *spicata* | MF349570 | MF350062 | MF348604 |  |
| AD3HK37 | [Van Neste 431](http://n2t.net/ark:/65665/3b23b587a-bb92-4faf-95db-30349bd28ac1) | Liliaceae | *Polygonatum* | *biflorum* | MF349215 | MF350061 | MF348606 |  |
| AD7LN77 | [Van Neste 712](http://n2t.net/ark:/65665/3c788226e-1f02-4e6d-9d14-a72564fc2fab) | Liliaceae | *Trillium* | *underwoodii* | MF349652 | MF350123 | MF348517 | MF348958 |
| AD5JT05 | [Van Neste 695](http://n2t.net/ark:/65665/3a23f0bd8-0f1b-4887-8b31-84520ebe1804) | Liliaceae | *Zigadenus* | *glaberrimus* | MF349683 | MF350145 |  |  |
| AD5JT18 | [Van Neste 645](http://n2t.net/ark:/65665/385e81192-b021-450d-b5b0-7a5cb31bfc10) | Linaceae | *Linum* | *perenne* | MF349746 | MF350178 | MF348420 | MF348896 |
| AD3HI53 | [Van Neste 247](http://n2t.net/ark:/65665/3084dc190-0544-4e3b-983b-c71d1c81129f) | Lycopodiaceae | *Huperzia* | *tetrasticha* | MF349529 |  | MF348649 |  |
| AD5JS70 | [Van Neste 623](http://n2t.net/ark:/65665/32c98d975-33d5-48ec-8810-a878bac6ca27) | Lycopodiaceae | *Lycopodium* | *obscurum* | MF349698 |  | MF348470 |  |
| AB6QQ16 | [Van Neste 116](http://n2t.net/ark:/65665/30661dc80-ea39-4f6c-93b9-2433898183f9) | Lycopodiaceae | *Phlegmariurus* | *salvinioides* | MF349694 |  | MF348474 |  |
| AD7LJ01 | [Gostel 151](http://n2t.net/ark:/65665/3b690dd81-8b0a-4555-bfa0-36b4864b5403) | Lythraceae | *Lawsonia* | *inermis* | MF349463 | MF349989 | MF348706 | MF349089 |
| AD0EE81 | [Van Neste 358](http://n2t.net/ark:/65665/3e1f10fe5-0b67-44f0-a1cc-f3cda9904dcb) | Lythraceae | *Lythrum* | *salicaria* | MF349292 | MF350260 | MF348392 |  |
| AD7LN72 | [Van Neste 717](http://n2t.net/ark:/65665/3f3d10414-bfd5-42b6-bcf8-0929a89c6bf0) | Magnoliaceae | *Magnolia* | *ashei* | MF349627 | MF350101 | MF348545 |  |
| AD0EG00 | [Van Neste 467](http://n2t.net/ark:/65665/3944c318f-aed4-4e62-b859-228b0db683ca) | Magnoliaceae | *Magnolia* | *kobus* | MF349801 | MF350223 | MF348355 |  |
| AD5JS57 | [Van Neste 610](http://n2t.net/ark:/65665/353e908cb-8a3a-4142-9155-863fd2c6679f) | Magnoliaceae | *Magnolia* | *virginiana* | MF349720 | MF350161 | MF348448 |  |
| AD7LK07 | [Gostel 166](http://n2t.net/ark:/65665/38f380178-ba0a-4458-90f4-04f9bdb5cd51) | Malpighiaceae | *Galphimia* | *gracilis* | MF349245 | MF349853 |  | MF349119 |
| AD5JQ98 | [Van Neste 584](http://n2t.net/ark:/65665/3e0a5381b-c196-40a8-a354-94436d768488) | Malpighiaceae | *Tristellateia* | *australasiae* | MF349685 | MF350152 | MF348468 | MF348928 |
| AD7LK03 | [Gostel 162](http://n2t.net/ark:/65665/343c925bb-49ba-4ec5-b903-04b3b2309a08) | Malvaceae | *Abelmoschus* | *moschatus* | MF349247 | MF349855 | MF348726 | MF349102 |
| AG2KL59 | [Vo 93](http://n2t.net/ark:/65665/35e97d3f2-28c8-44a1-a56f-eab9ea2b0aed) | Malvaceae | *Alcea* | *rosea* | MF349311 | MF349851 | MF348839 | MF349204 |
| AD5JT21 | [Van Neste 648](http://n2t.net/ark:/65665/35ac110af-5e3c-4c54-9354-28d137c53272) | Malvaceae | *Althaea* | *officinalis* | MF349736 | MF350173 | MF348431 | MF348903 |
| AD3HL08 | [Van Neste 504](http://n2t.net/ark:/65665/3f13f0b61-4a80-49df-b689-ec5547d3e19c) | Malvaceae | *Callirhoe* | *involucrata* | MF349521 | MF350025 | MF348655 | MF349059 |
| AG2KL51 | [Vo 89](http://n2t.net/ark:/65665/3ac79857d-54d8-44a3-a553-135e56517084) | Malvaceae | *Malva* | *sylvestris* | MF349342 | MF349879 |  | MF349177 |
| AD3HI82 | [Van Neste 276](http://n2t.net/ark:/65665/3a58c55d3-9fad-4368-a97f-6412ba03baf6) | Malvaceae | *Pavonia* | *multiflora* | MF349536 | MF350034 | MF348641 | MF349048 |
| AD0EF72 | [Van Neste 451](http://n2t.net/ark:/65665/3a7ed2ded-4cfb-4e01-aa98-f0c8706020b4) | Malvaceae | *Sida* | *fallax* | MF349766 | MF350256 | MF348403 | MF348884 |
| AD3HL97 | [Van Neste 451](http://n2t.net/ark:/65665/3a7ed2ded-4cfb-4e01-aa98-f0c8706020b4) | Malvaceae | *Sida* | *fallax* | MF349586 | MF350072 | MF348585 | MF349000 |
| AG2KL63 | [Vo 95](http://n2t.net/ark:/65665/347ac3827-e705-4739-a1fd-5abdbc49ec12) | Marantaceae | *Calathea* | *lancifolia* | MF349349 | MF349883 |  |  |
| AD3HK28 | [Van Neste 422](http://n2t.net/ark:/65665/30bcde76f-7dab-43f9-84d6-fd68a2fe96eb) | Melanthiaceae | *Trillium* | *luteum* | MF349605 | MF350086 | MF348566 | MF348984 |
| AD0ED86 | [Van Neste 279](http://n2t.net/ark:/65665/3c414b517-74bc-4f42-b152-9a50ada48f6b) | Melastomataceae | *Medinilla* | *cummingii* | MF349774 | MF350200 | MF348390 | MF348879 |
| AD7LJ02 | [Gostel 150](http://n2t.net/ark:/65665/3c32fadeb-5e18-4760-a874-9b8f9d06c52e) | Meliaceae | *Azadirachta* | *indica* | MF349454 | MF349980 |  |  |
| AD0EE87 | [Van Neste 365](http://n2t.net/ark:/65665/32abe5e1d-670e-45a7-b529-0af2b63b9abb) | Moraceae | *Morus* | *alba* | MF349804 | MF350225 | MF348351 | MF348856 |
| AD7LJ04 | [Gostel 153](http://n2t.net/ark:/65665/3e3225a4f-b903-48fb-8208-153a8dd9cd98) | Moringaceae | *Moringa* | *oleifera* | MF349444 | MF349970 | MF348725 |  |
| AG2KL67 | [Ingram 12](http://n2t.net/ark:/65665/35c84243c-f1f0-4d33-9367-3f6c7e91db21) | Myricaceae | *Morella* | *pensylvanica* | MF349373 | MF349904 | MF348791 | MF349160 |
| AC9DD47 | [Van Neste 246](http://n2t.net/ark:/65665/366e57100-e781-4854-b7f9-dca87f42d767) | Nepenthaceae | *Nepenthes* | *fusca* | MF349782 | MF350208 | MF348377 |  |
| AD5JP98 | [Kelloff 1615](http://n2t.net/ark:/65665/34f2856f3-993d-4f11-9717-4311cc161ca0) | Nyctaginaceae | *Mirabilis* | *nyctaginea* | MF349785 | MF350210 | MF348375 | MF348874 |
| AG2KM33 | [Sedaghatpour 2016-82](http://n2t.net/ark:/65665/3502fa7d6-8040-4b61-aad7-a77b4cd84ee6) | Nymphaeaceae | *Nuphar* | *lutea* | MF349225 | MF349884 | MF348808 | MF349174 |
| AD7LI69 | [Kelloff 1631](http://n2t.net/ark:/65665/3362d920b-d603-4dfc-a205-dfcf7492dd3f) | Nyssaceae | *Nyssa* | *sylvatica* | MF349555 | MF350049 | MF348622 | MF349032 |
| AD7LM83 | [Vo 79](http://n2t.net/ark:/65665/3539040a1-f22d-4031-bfa7-4b34f2328aff) | Oleaceae | *Fraxinus* | *pennsylvanica* | MF349421 | MF349949 | MF348749 | MF349122 |
| AG2KL57 | [Vo 92](http://n2t.net/ark:/65665/39c77e85f-55a4-4eea-8e19-5c4c816f1dae) | Oleaceae | *Jasminum* | *sambac* | MF349321 | MF349862 | MF348830 | MF349197 |
| AD7LM55 | [Sedaghatpour 2016-47](http://n2t.net/ark:/65665/3c9af5c7c-297c-48b5-b077-5b95f1c1b9af) | Oleaceae | *Osmanthus* | *heterophyllus* | MF349406 | MF349932 | MF348766 | MF349135 |
| AD7LH19 | [Van Neste 742](http://n2t.net/ark:/65665/362747b52-6e9c-4e99-9ed9-0ad6fd6b08d7) | Onagraceae | *Circaea* | *canadensis* | MF349680 |  |  | MF348941 |
| AG2KK47 | [Vo 83](http://n2t.net/ark:/65665/33dd6ccc0-1d72-4822-82c0-2c14d61989a8) | Onagraceae | *Gaura* | *lindheimeri* | MF349387 | MF349914 |  | MF349149 |
| AD7LI74 | [Kelloff 1636](http://n2t.net/ark:/65665/31dd3f7e4-6a46-4462-974f-1d7d9607b200) | Onagraceae | *Ludwigia* | *alternifolia* | MF349608 | MF350274 | MF348564 |  |
| AD7LN88 | [Van Neste 701](http://n2t.net/ark:/65665/3b691ec43-e9a2-4998-9e1d-c853a18eca45) | Onagraceae | *Oenothera* | *biennis* | MF349644 |  |  | MF348963 |
| AG2KL81 | [Ingram 19](http://n2t.net/ark:/65665/3ffb2206f-8a99-4d0c-8d50-e1602375f02b) | Onocleaceae | *Matteuccia* | *struthipteris* | MF349310 |  |  |  |
| AD7LM00 | [Vo 68](http://n2t.net/ark:/65665/3f004749e-bb47-4555-8944-932c51f1f35d) | Orchidaceae | *Aerangis* | *hariotiana* | MF349460 | MF349987 | MF348708 |  |
| AD3HI44 | [Van Neste 238](http://n2t.net/ark:/65665/33b6c3bdd-2066-4c6b-af72-780b55dea2bf) | Orchidaceae | *Aeranthes* | *grandidierana* | MF349518 | MF350023 |  | MF349061 |
| AD7LN17 | [Faulconer 56](http://n2t.net/ark:/65665/3b3abeef9-b90c-4f3f-8217-3fa62b5ae31e) | Orchidaceae | *Aerides* | *falcata* |  | MF349939 | MF348759 |  |
| AD3HI45 | [Van Neste 239](http://n2t.net/ark:/65665/39c0f1ffb-5e81-4732-9ad1-1d6aa734a1b4) | Orchidaceae | *Brassia* | *signata* | MF349508 | MF350015 |  |  |
| AD7LM67 | [Sedaghatpour 2016-53](http://n2t.net/ark:/65665/30226cbb0-6e25-45a1-a902-5f7208f92d30) | Orchidaceae | *Calanthe* | *discolor* | MF349422 | MF349950 |  | MF349121 |
| AD7LN43 | [Faulconer 69](http://n2t.net/ark:/65665/3c9e1d2d6-ec9b-45de-b5e2-f752084698b2) | Orchidaceae | *Cochleanthes* | *amazonica* x *sib* | MF349445 | MF349971 | MF348724 | MF349101 |
| AD3HI47 | [Van Neste 241](http://n2t.net/ark:/65665/36d5b5b98-1d04-4b33-9e6b-e60c27aaa669) | Orchidaceae | *Cynorkis* | *fastigiata* | MF349496 | MF350008 |  | MF349073 |
| AD3HI50 | [Van Neste 244](http://n2t.net/ark:/65665/36ef64ebf-8093-4a7c-b84a-0563b3c52b76) | Orchidaceae | *Dendrobium* | *lindleyi* | MF349469 | MF349993 |  | MF349084 |
| AD7LN35 | [Faulconer 65](http://n2t.net/ark:/65665/3b912b774-e4bc-46e1-b69f-f393e03be69f) | Orchidaceae | *Domingoa* | *purpurea* | MF349411 | MF349938 | MF348760 |  |
| AD7LJ68 | [Vo 25](http://n2t.net/ark:/65665/38fb1e747-1de2-4b07-8269-c2ad8d175ed2) | Orchidaceae | *Elleanthus* | *purpureus* | MF349453 | MF349979 | MF348714 | MF349098 |
| AD3HI48 | [Van Neste 242](http://n2t.net/ark:/65665/3081d629b-8f15-434b-aaf8-bd02742a1362) | Orchidaceae | *Encyclia* | *alata* | MF349485 | MF350003 |  |  |
| AD7LM02 | [Vo 69](http://n2t.net/ark:/65665/31a738a5d-1df8-4702-9c7c-11a0cdfb2442) | Orchidaceae | *Eulophia* | *macra* | MF349451 |  | MF348716 | MF349099 |
| AD7LM08 | [Vo 72](http://n2t.net/ark:/65665/3a5cff53a-974d-456b-a203-a56d49ae0843) | Orchidaceae | *Grammatophyllum* | *scriptum* var. *citrinum* |  | MF349952 | MF348746 |  |
| AD7LN49 | [Faulconer 72](http://n2t.net/ark:/65665/37acf8e31-c738-4fb9-b670-bebed6226a5e) | Orchidaceae | *Grosourdya* | *appendiculata* |  | MF349955 | MF348744 |  |
| AD7LJ66 | [Vo 24](http://n2t.net/ark:/65665/325d73683-719c-42b5-ad84-f9ae3a7d989e) | Orchidaceae | *Isochilus* | *linearis* | MF349462 |  | MF348707 | MF349090 |
| AD7LM04 | [Vo 70](http://n2t.net/ark:/65665/31664e688-2731-4d6e-8f80-23b99cf5e2e3) | Orchidaceae | *Laelia* | *lueddemannii* | MF349442 | MF349968 | MF348728 |  |
| AD7LN37 | [Faulconer 66](http://n2t.net/ark:/65665/3aef40772-9448-4cbb-a72e-6c196e2580f9) | Orchidaceae | *Maxillaria* | *splendens* |  |  | MF348771 |  |
| AD3HI43 | [Van Neste 237](http://n2t.net/ark:/65665/3a483ad4a-b890-4cac-b0b2-37849093d29b) | Orchidaceae | *Maxillariella* | *tenuifolia* | MF349530 | MF350265 | MF348843 | MF349053 |
| AD7LN21 | [Faulconer 58](http://n2t.net/ark:/65665/37f1b8598-5a00-483c-9412-dd9e793adfbd) | Orchidaceae | *Neohenricia* | *sibbettii* | MF349392 | MF349919 | MF348779 | MF349147 |
| AD7LN29 | [Faulconer 62](http://n2t.net/ark:/65665/33631c48a-6e7c-4c03-b18b-67a379d1527f) | Orchidaceae | *Ornithogalum* | *perdurans* | MF349427 | MF349956 |  | MF349118 |
| AD7LM06 | [Vo 71](http://n2t.net/ark:/65665/32ed8d71e-0526-4fcc-9628-643603499116) | Orchidaceae | *Peristeria* | *elata* |  | MF349960 | MF348738 | MF349114 |
| AD7LN39 | [Faulconer 67](http://n2t.net/ark:/65665/35fa81764-d600-4793-8235-3eef4cf2ab8f) | Orchidaceae | *Pholidota* | *pallida* | MF349391 | MF349918 | MF348780 |  |
| AD7LJ60 | [Vo 21](http://n2t.net/ark:/65665/374785288-580b-462e-871a-63bf49a69a1d) | Orchidaceae | *Restrepia* | spp. | MF349217 | MF349936 |  | MF349131 |
| AD7LN45 | [Faulconer 70](http://n2t.net/ark:/65665/312cba58d-5a3d-4214-a5b6-e17c9eee34e0) | Orchidaceae | *Rhetinantha* | *notylioglossa* | MF349435 | MF349962 | MF348734 | MF349111 |
| AG2KJ80 | [Faulconer 75](http://n2t.net/ark:/65665/36ec0695d-5a97-40a3-9729-0be56d60814f) | Orchidaceae | *Sievekingia* | *harrenhusana* | MF349410 | MF349937 | MF348761 | MF349130 |
| AD3HI40 | [Van Neste 234](http://n2t.net/ark:/65665/3e9b812fe-c331-42e3-ae9f-f435e2f2efba) | Orchidaceae | *Stanhopea* | *oculata* | MF349522 |  |  |  |
| AD7LJ64 | [Vo 23](http://n2t.net/ark:/65665/3e1277bfb-391d-4e58-9599-0eaeeb095090) | Orchidaceae | *Stelis* | *cylindrica* | MF349401 | MF349928 | MF348772 | MF349139 |
| AD7LN33 | [Faulconer 64](http://n2t.net/ark:/65665/3dab88899-2ade-4db9-b121-a02767d2d781) | Orchidaceae | *Stelis* | *quadrifida* | MF349418 | MF349946 |  |  |
| AD3HI49 | [Van Neste 243](http://n2t.net/ark:/65665/30342207f-3c10-41ac-8391-2b2aee32f4ee) | Orchidaceae | *Tetramicra* | *canaliculata* | MF349477 | MF349998 |  |  |
| AD7LN53 | [Faulconer 74](http://n2t.net/ark:/65665/3a505c4d4-39cf-4814-b585-a857d79bd1db) | Orchidaceae | *Thrixspermum* | *raciborskii* |  | MF349945 | MF348752 |  |
| AD3HI42 | [Van Neste 236](http://n2t.net/ark:/65665/3c1c5026f-8b02-4fd4-8225-06266373ec89) | Orchidaceae | *Thunia* | *alba* | MF349537 | MF350035 |  | MF349047 |
| AD7LN41 | [Faulconer 68](http://n2t.net/ark:/65665/3bfcf882b-f398-46b8-a128-496c7d5242e9) | Orchidaceae | *Trichoglottis* | *seidenfadenii* |  | MF349981 | MF348713 | MF349097 |
| AD7LN27 | [Faulconer 61](http://n2t.net/ark:/65665/3b9cc07cc-009f-4c35-9598-7306bb8acfed) | Orchidaceae | *Vanda* | *limbata* x *testacea* |  | MF349822 | MF348733 | MF349110 |
| AD7LN25 | [Faulconer 60](http://n2t.net/ark:/65665/38edb2270-8fde-4952-afba-2cd0fa00471f) | Orchidaceae | *Vanilla* | *planifolia* | MF349446 | MF349972 | MF348723 |  |
| AD7LI36 | [Van Neste 811](http://n2t.net/ark:/65665/313b9ac3a-b2a3-45e6-b150-374edabc3a19) | Oxalidaceae | *Averrhoa* | *carambola* | MF349621 | MF350097 | MF348551 | MF348978 |
| AD0EF87 | [Van Neste 458](http://n2t.net/ark:/65665/379c8a515-38bf-47cb-b7d6-16fe452ce6bb) | Oxalidaceae | *Oxalis* | *florida* | MF349228 | MF350282 | MF348354 | MF348858 |
| AD5JP54 | [Van Neste 556](http://n2t.net/ark:/65665/33f88078b-5f7e-4cc4-ae8d-25951a82c211) | Oxalidaceae | *Oxalis* | *florida* | MF349759 | MF350188 | MF348408 |  |
| AD5JT28 | [Van Neste 655](http://n2t.net/ark:/65665/3f382ca50-e7f4-405c-8e06-9fc20033d773) | Papaveraceae | *Corydalis* | *ochroleuca* | MF349688 | MF350146 | MF348481 |  |
| AD7LM43 | [Gostel 179](http://n2t.net/ark:/65665/3fb07b317-859f-4e7b-bfc1-e2b8f96c7fe6) | Papaveraceae | *Dicentra* | spp. | MF349220 | MF349976 | MF348718 |  |
| AG2KK85 | [Marcelli 14](http://n2t.net/ark:/65665/39d543f7b-2559-4b7f-a59b-171949a0ab36) | Papaveraceae | *Eschscholzia* | *californica* | MF349324 | MF349864 | MF348827 | MF349195 |
| AG2KM21 | [Marcelli 24](http://n2t.net/ark:/65665/3a57a23b6-5f69-4faf-b66e-b339e01f38a9) | Papaveraceae | *Macleaya* | *cordata* | MF349380 | MF349911 |  | MF349155 |
| AD3HL38 | [Van Neste 534](http://n2t.net/ark:/65665/38d1f768a-756e-4155-9d87-fe011611d05b) | Papaveraceae | *Stylophorum* | *diphyllum* | MF349655 | MF350125 | MF348514 | MF348955 |
| AD3HL03 | [Van Neste 499](http://n2t.net/ark:/65665/35be91de9-da54-4878-a0ed-670883676698) | Phrymaceae | *Mimulus* | *ringens* | MF349567 | MF350058 | MF348608 | MF349017 |
| AD3HL25 | [Van Neste 521](http://n2t.net/ark:/65665/341362977-58c1-498e-ac81-c7e08ffb1eec) | Phrymaceae | *Mimulus* | *ringens* | MF349630 | MF350104 | MF348542 | MF348974 |
| AG2KK71 | [Marcelli 7](http://n2t.net/ark:/65665/368e6226b-9817-4572-b4fc-7604c3a7a7e0) | Phytolaccaceae | *Petiveria* | *alliaceae* | MF349376 | MF349907 | MF348789 | MF349158 |
| AD0EF15 | [Van Neste 386](http://n2t.net/ark:/65665/3c634a65d-7d9c-4f84-ba80-58e207188b2d) | Phytolaccaceae | *Phytolacca* | *americana* | MF349796 | MF350220 |  | MF348863 |
| AD0EF15 | [Van Neste 386](http://n2t.net/ark:/65665/3c634a65d-7d9c-4f84-ba80-58e207188b2d) | Phytolaccaceae | *Phytolacca* | *americana* |  |  | MF348361 |  |
| AD3HK60 | [Van Neste 457](http://n2t.net/ark:/65665/34a6dba3f-51d0-4973-ae37-af784421cb1c) | Pinaceae | *Glyptostrobus* | *pensilis* | MF349544 |  |  | MF349042 |
| AD7LH38 | [Van Neste 762](http://n2t.net/ark:/65665/36f784672-1eaa-4054-8db0-725ff3ecb903) | Pinaceae | *Juniperus* | *virginiana* | MF349613 |  | MF348509 | MF348953 |
| AD3HL21 | [Van Neste 517](http://n2t.net/ark:/65665/3e3c1bfcb-7740-426b-9c89-61d4301a5706) | Pinaceae | *Larix* | *laricina* | MF349656 |  | MF348513 | MF348954 |
| AD7LH86 | [Van Neste 781](http://n2t.net/ark:/65665/3f15bab63-9e44-4338-ba4f-1c39a532fca2) | Pinaceae | *Metasequoia* | *glyptostroboides* | MF349632 |  | MF348539 | MF348973 |
| AD3HK69 | [Van Neste 465](http://n2t.net/ark:/65665/3d3b03daf-8533-4bb8-ae2a-7c32d011b0d6) | Pinaceae | *Picea* | *glauca* | MF349560 |  | MF348617 | MF349026 |
| AD3HJ29 | [Van Neste 323](http://n2t.net/ark:/65665/3fddd0c3a-d1c8-49b8-b2d2-c1e1bc59dbb9) | Pinaceae | *Picea* | *wilsonii* | MF349465 |  | MF348703 | MF349087 |
| AD3HK68 | [Van Neste 464](http://n2t.net/ark:/65665/38f485c68-fdf8-4c8d-88e7-6d608e561812) | Pinaceae | *Pinus* | *armandii* |  |  |  | MF349015 |
| AD7LH42 | [Van Neste 766](http://n2t.net/ark:/65665/3eca27f82-6e5d-45f9-bc91-0b55d6c0cdec) | Pinaceae | *Pinus* | *virginiana* |  |  |  | MF348972 |
| AD3HJ15 | [Van Neste 310](http://n2t.net/ark:/65665/351b4f8a3-6d1d-41aa-a754-3a7dd8a51cfb) | Pinaceae | *Platycladus* | *orientalis* | MF349466 |  |  | MF349086 |
| AD7LH82 | [Van Neste 777](http://n2t.net/ark:/65665/32959c420-dd41-40c3-b475-1a0cb0417445) | Pinaceae | *Sciadopitys* | *verticillata* | MF349649 |  |  | MF348961 |
| AB6QQ34 | [Van Neste 135](http://n2t.net/ark:/65665/3c488d978-5b86-4423-b74d-9396e1ec90f4) | Pinaceae | *Taiwania* | *cryptomerioides* | MF349732 |  | MF348434 |  |
| AD7LN71 | [Van Neste 718](http://n2t.net/ark:/65665/312dab0e2-0885-4b03-9276-b79061932479) | Pinaceae | *Taxodium* | *iascendens* | MF349489 |  |  | MF349075 |
| AD3HK58 | [Van Neste 455](http://n2t.net/ark:/65665/39b73b046-9f6d-4711-8e57-9c7cdc4f8a77) | Pinaceae | *Taxodium* | *distichum* |  |  |  | MF349025 |
| AD3HK63 | [Van Neste 459](http://n2t.net/ark:/65665/36bf2e5d6-301e-4159-a7e6-5056d937f361) | Pinaceae | *Thuja* | *orientalis* | MF349266 |  |  | MF348985 |
| AD3HK27 | [Van Neste 421](http://n2t.net/ark:/65665/3f49123ef-32e5-4823-a26c-dc1f0b9fce5c) | Pinaceae | *Tsuga* | *canadensis* | MF349553 |  | MF348625 | MF349035 |
| AC9DD18 | [Van Neste 217](http://n2t.net/ark:/65665/35b30ef4e-2470-4fe9-86eb-120e31acba74) | Piperaceae | *Peperomia* | *tetraphylla* | MF349799 |  |  | MF348860 |
| AD7LH77 | [Van Neste 772](http://n2t.net/ark:/65665/3f848f89c-b6dc-41ff-8727-131b21f0c5f9) | Plantaginaceae | *Chelone* | *lyonii* | MF349668 | MF350135 | MF348499 |  |
| AD5JQ07 | [Kelloff 1624](http://n2t.net/ark:/65665/33b38b284-24da-4b46-bd79-8d9e5d84936f) | Plantaginaceae | *Gratiola* | *viscidula* | MF349290 | MF350197 | MF348396 | MF348881 |
| AD0EF94 | [Van Neste 470](http://n2t.net/ark:/65665/39d088242-33da-4e9a-9d3c-ec888cc1f61b) | Plantaginaceae | *Plantago* | *lanceolata* | MF349765 | MF350193 |  |  |
| AD0EF94 | [Van Neste 470](http://n2t.net/ark:/65665/39d088242-33da-4e9a-9d3c-ec888cc1f61b) | Plantaginaceae | *Plantago* | *lanceolata* |  |  |  | MF348885 |
| AD0EG80 | [Van Neste 546](http://n2t.net/ark:/65665/359ed61a3-e0cf-4b4e-b646-14406a3e6d4a) | Plantaginaceae | *Russelia* | *equisetiformis* | MF349793 | MF350218 | MF348365 | MF348866 |
| AD7LH94 | [Van Neste 789](http://n2t.net/ark:/65665/31872d92b-7478-424d-96a1-1f56184a9608) | Plumbaginaceae | *Ceratostigma* | *plumbaginoides* | MF349658 |  | MF348511 |  |
| AD3HK75 | [Van Neste 471](http://n2t.net/ark:/65665/3665cc098-d5a7-4947-bc30-3c63a9b17091) | Poaceae | *Agrostis* | *perennans* | MF349265 |  | MF348568 | MF348986 |
| AD3HJ83 | [Van Neste 377](http://n2t.net/ark:/65665/367f23ac4-b131-489d-9424-04236c0a5890) | Poaceae | *Arthraxon* | *hispidus* | MF349579 | MF350067 | MF348593 | MF349008 |
| AD5JQ53 | [Kelloff 1592](http://n2t.net/ark:/65665/362c6d817-e2d7-44b3-86a0-7410af62e3d0) | Poaceae | *Cenchrus* | *incertus* | MF349556 |  | MF348621 | MF349030 |
| AD3HK67 | [Van Neste 463](http://n2t.net/ark:/65665/3de1bc263-b00a-4640-b979-43e52066c0dc) | Poaceae | *Deschampsia* | *cespitosa* | MF349576 |  | MF348597 | MF349010 |
| AD5JQ62 | [Kelloff 1601](http://n2t.net/ark:/65665/36d2c901b-9dae-42f8-98a8-0b26019e3b0a) | Poaceae | *Dichanthelium* | *laxiflorum* | MF349564 |  | MF348611 | MF349020 |
| AD7LI81 | [Kelloff 1643](http://n2t.net/ark:/65665/391b3a629-2881-4306-8943-17aedb1bf698) | Poaceae | *Echinochloa* | *muricata* | MF349589 |  | MF348582 | MF348998 |
| AD0EE85 | [Van Neste 362](http://n2t.net/ark:/65665/38467c47d-ed27-45be-9f4a-7be786749804) | Poaceae | *Elymus* | *macgregorii* | MF349808 | MF350281 | MF348342 | MF348852 |
| AD3HJ68 | [Van Neste 362](http://n2t.net/ark:/65665/38467c47d-ed27-45be-9f4a-7be786749804) | Poaceae | *Elymus* | *macgregorii* | MF349479 |  | MF348691 | MF349077 |
| AD7LI75 | [Kelloff 1637](http://n2t.net/ark:/65665/3e6f7cfff-ad49-4327-adc7-7597e9c035b4) | Poaceae | *Eragrostis* | *spectabilis* | MF349599 | MF350080 | MF348574 | MF348991 |
| AB6QQ29 | [Van Neste 130](http://n2t.net/ark:/65665/32a58547b-61fe-44ee-8a17-494380fcca2d) | Poaceae | *Fargesia* | *rufa* | MF349281 | MF350149 | MF348475 |  |
| AD7LK05 | [Gostel 164](http://n2t.net/ark:/65665/32edeb451-01a9-4686-bcc0-13850b3f248d) | Poaceae | *Hakonechloa* | *macra* | MF349246 | MF349854 | MF348736 |  |
| AD7LN55 | [Van Neste 730](http://n2t.net/ark:/65665/3a0d18e6e-b751-43a8-90e8-8fbc702ca084) | Poaceae | *Panicum* | *virgatum* | MF349681 |  | MF348486 | MF348940 |
| AD7LI01 | [Van Neste 796](http://n2t.net/ark:/65665/36dfadc23-2a08-4689-9d50-118ece923ae6) | Poaceae | *Paspalum* | *floridanum* | MF349614 |  | MF348558 | MF348981 |
| AD3HJ99 | [Van Neste 393](http://n2t.net/ark:/65665/351f4cc4b-dd68-47f9-95c6-f7d3d033a65b) | Poaceae | *Setaria* | *faberi* | MF349597 |  |  | MF348992 |
| AD7LH35 | [Van Neste 758](http://n2t.net/ark:/65665/34bd8e1a5-7428-498e-93a9-f9efa442898a) | Poaceae | *Tripsacum* | *dactyloides* | MF349679 |  | MF348487 |  |
| AD3HL35 | [Van Neste 531](http://n2t.net/ark:/65665/3d549c927-1296-40a3-bed5-3490b35422ed) | Poaceae | *Uniola* | *latifolia* | MF349666 | MF350133 | MF348503 |  |
| AD3HJ27 | [Van Neste 321](http://n2t.net/ark:/65665/370dc193e-b674-4f03-9cb9-c3b89ac1fc56) | Podocarpaceae | *Podocarpus* | *macrophyllus* | MF349481 |  | MF348688 |  |
| AD0EG56 | [Van Neste 515](http://n2t.net/ark:/65665/3658249f9-5723-42be-aaf2-93970eda1938) | Polemoniaceae | *Phlox* | *paniculata* | MF349786 | MF350262 | MF348374 | MF348873 |
| AD3HJ48 | [Van Neste 342](http://n2t.net/ark:/65665/3dea76b0d-29e3-4f1c-8f0b-10b3237fa472) | Polygonaceae | *Bistorta* | *amplexicaulis* | MF349524 |  | MF348654 | MF349057 |
| AD0EF13 | [Van Neste 413](http://n2t.net/ark:/65665/30c0d6963-d014-4c26-8d5a-9c6256cb8549) | Polygonaceae | *Persicaria* | *amphibia* |  |  | MF348381 |  |
| AD0EG07 | [Van Neste 476](http://n2t.net/ark:/65665/387bdb394-6594-4d49-a9a7-a01861bccd5e) | Polygonaceae | *Persicaria* | *longiseti* | MF349232 | MF349821 | MF348332 | MF348848 |
| AD3HK80 | [Van Neste 476](http://n2t.net/ark:/65665/387bdb394-6594-4d49-a9a7-a01861bccd5e) | Polygonaceae | *Persicaria* | *longiseta* | MF349259 | MF350059 |  | MF349016 |
| AD7LH18 | [Van Neste 741](http://n2t.net/ark:/65665/371c8e505-de03-46b8-928c-864447fcaa3d) | Polygonaceae | *Persicaria* | *virginiana* | MF349268 | MF350092 | MF348556 |  |
| AD5JS59 | [Van Neste 612](http://n2t.net/ark:/65665/316758286-ab4f-4eee-b085-5ad1a96de93b) | Polygonaceae | *Polygonum* | *sagittatum* | MF349283 | MF349839 | MF348469 |  |
| AD0EF13 | [Van Neste 413](http://n2t.net/ark:/65665/30c0d6963-d014-4c26-8d5a-9c6256cb8549) | Polygonaceae | *Polygonum* | spp. | MF349207 | MF350283 |  |  |
| AD3HL55 | [Kelloff 1572](http://n2t.net/ark:/65665/3cf7d8fb3-d8ce-4809-99e4-54cf3962e164) | Polygonaceae | *Rumex* | *obtusifolius* | MF349550 |  | MF348629 |  |
| AD3HI55 | [Van Neste 249](http://n2t.net/ark:/65665/3b2b8cf54-2754-4708-bfb9-27546b2ba874) | Polypodiaceae | *Lecanopteris* | *crustacea* | MF349507 |  | MF348668 |  |
| AD7LI37 | [Van Neste 812](http://n2t.net/ark:/65665/3e40b1c30-50bd-4529-aaed-da8bdb8fa7b7) | Polypodiaceae | *Lemmaphyllum* | *microphyllum* | MF349267 |  | MF348559 |  |
| AD0EE00 | [Van Neste 290](http://n2t.net/ark:/65665/3d6e23603-7d2c-4632-8e0c-4eea94fc5632) | Polypodiaceae | *Microsorum* | *punctatum* | MF349779 |  | MF348380 |  |
| AD3HI96 | [Van Neste 290](http://n2t.net/ark:/65665/3d6e23603-7d2c-4632-8e0c-4eea94fc5632) | Polypodiaceae | *Microsorum* | *punctatum* | MF349475 |  | MF348696 |  |
| AD5JQ95 | [Van Neste 581](http://n2t.net/ark:/65665/333f756bb-34be-48e3-b79d-2a4da979e216) | Polypodiaceae | *Polypodium* | spp. | MF349224 |  | MF348446 |  |
| AD0EE92 | [Van Neste 370](http://n2t.net/ark:/65665/3e0b9f9e9-e905-47a3-98f3-25998f699ae6) | Pontederiaceae | *Pontederia* | *cordata* | MF349803 | MF350224 | MF348352 |  |
| AD5JS88 | [Van Neste 678](http://n2t.net/ark:/65665/37bcff249-23de-4026-8a5f-b751124db8dc) | Portulacaceae | *Anacampseros* | *crinita* | MF349734 | MF350171 | MF348433 |  |
| AD5JS96 | [Van Neste 686](http://n2t.net/ark:/65665/3b20f8ce2-cd40-41fd-b4f4-321b128e68f0) | Portulacaceae | *Avonia* | *papyracea* | MF349665 | MF350132 |  |  |
| AD5JT27 | [Van Neste 654](http://n2t.net/ark:/65665/37365833f-a449-414d-8e18-4ad012a7545a) | Portulacaceae | *Phemeranthus* | *teretifolius* | MF349696 | MF350150 | MF348472 |  |
| AD7LN89 | [Van Neste 674](http://n2t.net/ark:/65665/31aa7a4c3-fad4-43b1-8b61-b47accd773ad) | Primulaceae | *Lysimachia* | *ciliata* | MF349282 | MF349840 |  | MF348931 |
| AD3HJ41 | [Van Neste 335](http://n2t.net/ark:/65665/3edf85186-5c4b-49f1-93fd-742871d54d93) | Primulaceae | *Lysimachia* | *clethroides* | MF349478 |  | MF348694 | MF349078 |
| AC9DD60 | [Van Neste 259](http://n2t.net/ark:/65665/38b1bda8b-b042-4810-b8fd-a546681557d2) | Psilotaceae | *Psilotum* | *complanatum* | MF349812 |  | MF348337 |  |
| AC9DD51 | [Van Neste 250](http://n2t.net/ark:/65665/342fb7a0e-1334-42fe-8fe0-cb8189345f2f) | Pteridaceae | *Adiantum* | *hispidulum* | MF349770 |  | MF348397 |  |
| AD7LN11 | [Faulconer 53](http://n2t.net/ark:/65665/3dc83e275-c2a1-4406-b8fe-287939d81825) | Ranunculaceae | *Adonis* | *amurensis* | MF349436 | MF349963 | MF348732 | MF349109 |
| AD3HL01 | [Van Neste 497](http://n2t.net/ark:/65665/39fdd6ee2-f283-4013-8596-b115553763c9) | Ranunculaceae | *Anemone* | *virginiana* | MF349260 | MF350064 | MF348599 |  |
| AD7LN66 | [Van Neste 723](http://n2t.net/ark:/65665/38051aab0-48f7-4131-a3c2-c41f60baee1e) | Ranunculaceae | *Aquilegia* | *chrysantha* | MF349671 |  | MF348495 | MF348946 |
| AD7LN64 | [Van Neste 725](http://n2t.net/ark:/65665/320e9f990-0c91-45d0-8319-3752f5e64947) | Ranunculaceae | *Clematis* | *pitcheri* | MF349651 | MF350122 |  | MF348959 |
| AG2KL61 | [Vo 94](http://n2t.net/ark:/65665/32e4a4e26-5dbc-4618-9c81-039d30122120) | Ranunculaceae | *Consolida* | *ambigua* | MF349382 | MF350253 | MF348786 |  |
| AG2KL71 | [Ingram 14](http://n2t.net/ark:/65665/3afef691a-5864-49b7-846e-10268344c44e) | Ranunculaceae | *Coptis* | *japonica* var. *dissecta* | MF349351 | MF349886 |  |  |
| AD7LM12 | [Faulconer 28](http://n2t.net/ark:/65665/31bfb345f-d5dc-426e-88de-8ee3d3793c0a) | Ranunculaceae | *Helleborus* | *foetidus* | MF349408 | MF349934 | MF348764 | MF349134 |
| AG2KK89 | [Ingram 2](http://n2t.net/ark:/65665/32a92c96d-2b60-476a-b419-08c2029f31e3) | Ranunculaceae | *Hydrastis* | *canadensis* | MF349385 |  |  | MF349151 |
| AG2KK59 | [Marcelli 1](http://n2t.net/ark:/65665/3431c572c-270e-45a4-b566-bcf65b2161fa) | Ranunculaceae | *Nigella* | *damascena* | MF349345 | MF349880 | MF348810 | MF349176 |
| AD0EE49 | [Van Neste 336](http://n2t.net/ark:/65665/37198630f-219b-4ffe-8fe1-24f0dea70253) | Ranunculaceae | *Thalictrum* | *ichangense* | MF349819 | MF350239 | MF348328 | MF348847 |
| AD3HJ42 | [Van Neste 336](http://n2t.net/ark:/65665/37198630f-219b-4ffe-8fe1-24f0dea70253) | Ranunculaceae | *Thalictrum* | *ichangense* | MF349472 | MF349995 | MF348699 | MF349082 |
| AD5JT10 | [Van Neste 700](http://n2t.net/ark:/65665/3758075ae-c4ec-486a-8c26-f1282df7bc85) | Ranunculaceae | *Trautvetteria* | *caroliniensis* | MF349653 |  | MF348516 | MF348957 |
| AD3HL81 | [Van Neste 559](http://n2t.net/ark:/65665/397483dab-387a-4d1e-b57f-2e7fb6efda32) | Rapateaceae | *Stegolepis* | *guianensis* | MF349301 |  | MF348356 |  |
| AD0EG31 | [Van Neste 495](http://n2t.net/ark:/65665/3d052f339-cafc-4893-a8a5-dacfee8802da) | Rhamnaceae | *Ceanothus* | *americanus* | MF349773 | MF350198 | MF348394 | MF348880 |
| AG2KK45 | [Vo 82](http://n2t.net/ark:/65665/3d63b6fa5-f19f-4554-853b-889df412c54e) | Rosaceae | *Amelanchier* | *arborea* | MF349316 | MF350243 |  |  |
| AD7LL51 | [Sedaghatpour 2016-37](http://n2t.net/ark:/65665/32aad0ea8-48ad-4b88-bdc4-936ea77157af) | Rosaceae | *Aronia* | *arbutifolia* | MF349461 | MF349988 |  | MF349091 |
| AG2KK51 | [Vo 85](http://n2t.net/ark:/65665/36c59a1c9-68ec-4d51-a171-4be3a84d44ea) | Rosaceae | *Aruncus* | spp. | MF349242 | MF349908 |  |  |
| AD3HK65 | [Van Neste 461](http://n2t.net/ark:/65665/35c98e0ac-4e5f-4e18-9a7e-b70c0b841a41) | Rosaceae | *Cotoneaster* | *horizontalis* | MF349594 | MF350273 |  |  |
| AD3HJ09 | [Van Neste 303](http://n2t.net/ark:/65665/33adf4a39-2447-451c-808b-767081cc09d9) | Rosaceae | *Crataegus* | *monogyna* | MF349505 | MF350263 | MF348670 | MF349067 |
| AD3HJ78 | [Van Neste 372](http://n2t.net/ark:/65665/36f1a1da1-b088-441f-8036-c521e449ec55) | Rosaceae | *Crataegus* | *phaenopyrum* | MF349607 | MF350087 |  |  |
| AD3HJ33 | [Van Neste 327](http://n2t.net/ark:/65665/36aba2e32-18cf-45ca-ba12-cc7be1314ba3) | Rosaceae | *Filipendula* | *ulmaria* | MF349513 |  | MF348662 | MF349062 |
| AD3HL51 | [Kelloff 1568](http://n2t.net/ark:/65665/3de81c393-2ba7-4258-895d-7f4bd5b14f08) | Rosaceae | *Geum* | *canadense* | MF349574 |  | MF348600 | MF349011 |
| AG2KL73 | [Ingram 15](http://n2t.net/ark:/65665/337cea643-2a7a-4684-a4b3-e33b419376d8) | Rosaceae | *Kerria* | *japonica* | MF349341 |  |  |  |
| AD3HJ82 | [Van Neste 376](http://n2t.net/ark:/65665/3b12c4125-e6c1-4e14-8aaa-b0e79b71066a) | Rosaceae | *Malus* | *baccata* | MF349588 |  | MF348583 |  |
| AD3HK26 | [Van Neste 420](http://n2t.net/ark:/65665/35472b6df-397f-4587-a0b0-f7b96b9851e2) | Rosaceae | *Malus* | *coronaria* | MF349257 |  | MF348615 |  |
| AD3HJ81 | [Van Neste 375](http://n2t.net/ark:/65665/35b5c7a3f-9d2d-42a5-8e35-05cc41df057f) | Rosaceae | *Malus* | *floribunda* | MF349598 | MF350079 | MF348575 |  |
| AD3HK48 | [Van Neste 442](http://n2t.net/ark:/65665/34425326d-af90-4112-b482-0f6c6215ad60) | Rosaceae | *Neviusia* | *alabamensis* | MF349254 | MF350046 | MF348626 | MF349036 |
| AD7LM38 | [Faulconer 41](http://n2t.net/ark:/65665/3c5e85848-e4d1-463f-afb9-e50d67b8a1de) | Rosaceae | *Photinia* | *pyrofolia* | MF349397 | MF349924 |  | MF349143 |
| AD7LN62 | [Van Neste 727](http://n2t.net/ark:/65665/3b179561f-927c-47b6-86f6-cc9e0388ed52) | Rosaceae | *Physocarpus* | *opulifolius* | MF349634 | MF350108 | MF348536 |  |
| AD3HJ05 | [Van Neste 299](http://n2t.net/ark:/65665/3b9f599f1-3e8c-4238-a78c-9f562024c281) | Rosaceae | *Prunus* | *laurocerasus* | MF349527 |  | MF348651 | MF349054 |
| AD0EG19 | [Kelloff 1581](http://n2t.net/ark:/65665/308cc4f8b-7046-4190-8282-97b7a5aea5e8) | Rosaceae | *Prunus* | *serotina* | MF349231 |  | MF348364 | MF348865 |
| AD3HL64 | [Kelloff 1581](http://n2t.net/ark:/65665/308cc4f8b-7046-4190-8282-97b7a5aea5e8) | Rosaceae | *Prunus* | *serotina* | MF349591 |  | MF348581 |  |
| AD3HJ54 | [Van Neste 348](http://n2t.net/ark:/65665/3c81396d8-8ced-4e54-bde0-50c5de97e631) | Rosaceae | *Rhaphiolepis* | *delacourii* | MF349491 |  |  |  |
| AD3HJ13 | [Van Neste 307](http://n2t.net/ark:/65665/32adafdce-32da-4c69-a1da-3fedd9c288f4) | Rosaceae | *Rosa* | *setigera* | MF349476 | MF349997 | MF348695 | MF349079 |
| AD3HJ84 | [Van Neste 378](http://n2t.net/ark:/65665/32fa534a7-9ede-424b-af77-eb7e8f80d29b) | Rosaceae | *Rubus* | *laciniatus* | MF349569 |  | MF348605 |  |
| AD5JT40 | [Van Neste 668](http://n2t.net/ark:/65665/3e6e50026-cf86-42c7-b343-d15052184ecf) | Rosaceae | *Rubus* | *odoratus* | MF349716 | MF349835 | MF348451 | MF348915 |
| AD3HL61 | [Kelloff 1578](http://n2t.net/ark:/65665/319c4b74b-ac37-489d-9d68-ff20a797010e) | Rosaceae | *Rubus* | *phoenicolasius* | MF349611 |  | MF348561 | MF348982 |
| AD3HJ22 | [Van Neste 317](http://n2t.net/ark:/65665/3ad491353-9d64-458e-8d26-7bc8cdf4f4c4) | Rosaceae | *Spiraea* | *japonica* | MF349514 | MF350020 | MF348661 |  |
| AD7LM93 | [Faulconer 44](http://n2t.net/ark:/65665/38664e8f1-eab7-4f68-95f3-18b68b66bebe) | Rosaceae | *Spiraea* | *thunbergii* | MF349448 | MF349973 | MF348721 |  |
| AD7LI35 | [Van Neste 810](http://n2t.net/ark:/65665/36ffce2cf-d2d8-4725-a48c-3e83e8ac2234) | Rubiaceae | *Coffea* | *canephora* | MF349271 | MF350105 | MF348541 |  |
| AD5JQ52 | [Kelloff 1591](http://n2t.net/ark:/65665/32717663b-67d3-4b6c-a434-8f5337a5e3aa) | Rubiaceae | *Diodella* | *teres* | MF349565 | MF350056 | MF348610 | MF349019 |
| AD0EE83 | [Van Neste 360](http://n2t.net/ark:/65665/372f1fe41-7382-4ef9-81be-a63ce7cfdd7c) | Rubiaceae | *Diodia* | *virginiana* | MF349802 | MF350280 | MF348353 | MF348857 |
| AD3HJ66 | [Van Neste 360](http://n2t.net/ark:/65665/372f1fe41-7382-4ef9-81be-a63ce7cfdd7c) | Rubiaceae | *Diodia* | *virginiana* | MF349490 |  | MF348682 | MF349074 |
| AD7LH32 | [Van Neste 755](http://n2t.net/ark:/65665/3097947e4-e316-4391-a662-919fe882f764) | Rubiaceae | *Galium* | *circaezans* | MF349633 | MF349846 | MF348538 | MF348971 |
| AD5JQ69 | [Kelloff 1608](http://n2t.net/ark:/65665/313400527-9fef-4811-8837-5869b3338803) | Rubiaceae | *Galium* | *pilosum* | MF349609 | MF350088 | MF348563 | MF348983 |
| AG2KK23 | [Gostel 205](http://n2t.net/ark:/65665/3546908c2-725f-48e5-abfd-8f124bc89e6a) | Rubiaceae | *Gardenia* | *jasminoides* | MF349347 | MF349881 | MF348809 | MF349175 |
| AD5JT07 | [Van Neste 697](http://n2t.net/ark:/65665/333a93388-b79d-4999-85fb-9e10c9e1f7fb) | Rubiaceae | *Hamelia* | *patens* | MF349673 | MF350138 | MF348493 | MF348945 |
| AD3HI06 | [Van Neste 200](http://n2t.net/ark:/65665/3ae37d897-6e4e-47eb-84d2-eb6829f4c5a2) | Rubiaceae | *Hintonia* | *pulchra* | MF349548 | MF350042 | MF348632 | MF349039 |
| AD5JS52 | [Van Neste 605](http://n2t.net/ark:/65665/3abe3e19f-95d2-458c-8ed7-3c4421ca42c6) | Rubiaceae | *Mitchella* | *repens* |  |  |  | MF348935 |
| AD5JP70 | [Van Neste 572](http://n2t.net/ark:/65665/3401225df-15de-4e6a-ba33-35aaae318b03) | Rubiaceae | *Mussaenda* | *frondosa* | MF349700 | MF350153 | MF348467 | MF348927 |
| AD7LM63 | [Sedaghatpour 2016-51](http://n2t.net/ark:/65665/32fef0761-c598-4448-89de-30d96dd934fe) | Rutaceae | *Poncirus* | *trifoliata* | MF349439 | MF349965 |  | MF349106 |
| AG2KK67 | [Marcelli 5](http://n2t.net/ark:/65665/3d8fec1a9-7c2c-456f-a234-a1feb19326fe) | Rutaceae | *Zanthoxylum* | *clava-herculis* | MF349386 | MF350254 |  | MF349150 |
| AD0EE26 | [Van Neste 314](http://n2t.net/ark:/65665/353353140-a5ec-4154-bb1e-a80d3c3936c4) | Sapindaceae | *Aesculus* | *parviflora* | MF349307 | MF350240 | MF348327 | MF348846 |
| AD7LI68 | [Kelloff 1630](http://n2t.net/ark:/65665/3730ebd5e-2466-4624-8b36-f3bfbd83bd1d) | Sapindaceae | *Koelreuteria* | *paniculata* | MF349563 | MF350055 | MF348612 | MF349021 |
| AD7LN73 | [Van Neste 716](http://n2t.net/ark:/65665/3247112de-5d70-4989-bca0-198c5794eb52) | Sapindaceae | *Ungnadia* | *speciosa* | MF349635 |  | MF348535 | MF348970 |
| AC9DD09 | [Van Neste 206](http://n2t.net/ark:/65665/3f65d91e5-99a4-454c-a88d-a8596442667e) | Sarraceniaceae | *Sarracenia* | *leucophylla* | MF349790 | MF350214 | MF348369 | MF348870 |
| AG2KK11 | [Gostel 199](http://n2t.net/ark:/65665/3d103185f-0b2a-4f84-bb00-7eef7f730415) | Saururaceae | *Houttuynia* | *cordata* | MF349389 | MF349916 | MF348782 | MF349148 |
| AD3HL46 | [Van Neste 542](http://n2t.net/ark:/65665/3820117b7-4f6e-4ae2-9131-83105ac32a3f) | Saururaceae | *Saururus* | *cernuus* | MF349612 | MF350090 | MF348560 |  |
| AD5JS46 | [Van Neste 599](http://n2t.net/ark:/65665/38c5a7df3-cd17-40f9-a578-dfbde7723cfe) | Saururaceae | *Saururus* | *cernuus* | MF349730 | MF350167 | MF348437 |  |
| AD7LM75 | [Vo 75](http://n2t.net/ark:/65665/36894a6c8-7775-42d6-9812-af11f582436c) | Saxifragaceae | *Astilbe* | spp. | MF349235 | MF349985 |  | MF349095 |
| AD3HJ60 | [Van Neste 354](http://n2t.net/ark:/65665/379df3d66-7137-48e8-a7c9-2c9b97501163) | Saxifragaceae | *Deutzia* | *gracilis* | MF349523 | MF350026 | MF348844 | MF349058 |
| AD3HJ61 | [Van Neste 355](http://n2t.net/ark:/65665/3dae35ba1-5249-4b83-b6a9-4298034a64ee) | Saxifragaceae | *Hydrangea* | *serrata* | MF349512 | MF350019 | MF348663 | MF349063 |
| AD3HL28 | [Van Neste 524](http://n2t.net/ark:/65665/33441cdc1-ed6a-4a0d-a5cb-5d1a2af79953) | Saxifragaceae | *Itea* | *virginica* | MF349620 | MF350096 | MF348552 | MF348979 |
| AD3HJ10 | [Van Neste 304](http://n2t.net/ark:/65665/3927e7be7-8d81-41da-a0bf-a3945f548236) | Saxifragaceae | *Philadelphus* | *keteleerii* | MF349493 | MF350005 | MF348680 |  |
| AD7LI71 | [Kelloff 1633](http://n2t.net/ark:/65665/31178b374-6c81-412a-a51d-9870e905c1db) | Saxifragaceae | *Philadelphus* | *keteleerii* | MF349549 | MF350043 | MF348631 |  |
| AD0EE16 | [Van Neste 305](http://n2t.net/ark:/65665/304e35d22-f19a-4190-a988-3bb9a473195d) | Saxifragaceae | *Philadelphus* | *pekinensis* | MF349818 | MF350238 | MF348329 |  |
| AD3HJ11 | [Van Neste 305](http://n2t.net/ark:/65665/304e35d22-f19a-4190-a988-3bb9a473195d) | Saxifragaceae | *Philadelphus* | *pekinensis* | MF349482 | MF350001 | MF348687 |  |
| AD5JP50 | [Van Neste 552](http://n2t.net/ark:/65665/3c000a84a-0df6-475a-8a94-5bed069c01d8) | Saxifragaceae | *Ribes* | *odoratum* | MF349285 | MF350163 | MF348444 |  |
| AD7LN63 | [Van Neste 726](http://n2t.net/ark:/65665/3b3152141-6c99-4100-b07d-43ea2a98964c) | Saxifragaceae | *Ribes* | *odoratum* | MF349275 | MF350115 | MF348526 |  |
| AG2KK21 | [Gostel 204](http://n2t.net/ark:/65665/378b48be5-bb2a-4401-89b0-efa39caaa545) | Saxifragaceae | *Saxifraga* | *stolonifera* | MF349358 |  | MF348802 | MF349170 |
| AD7LM79 | [Vo 77](http://n2t.net/ark:/65665/3e589e799-25b4-4850-b52e-9ab1e52e4a59) | Saxifragaceae | *Tiarella* | *cordifolia* | MF349438 | MF349964 |  | MF349107 |
| AG2KL05 | [Ingram 10](http://n2t.net/ark:/65665/35cc4436e-f991-4458-b85d-58b9cfa84e9a) | Schisandraceae | *Illicium* | *floridanum* | MF349323 |  | MF348828 | MF349196 |
| AD0EE04 | [Van Neste 294](http://n2t.net/ark:/65665/3e1930992-79bb-4bcc-ad97-f9641a9fb72c) | Scrophulariaceae | *Buddleja* | *davidii* | MF349769 | MF350196 | MF348398 |  |
| AD5JS50 | [Van Neste 603](http://n2t.net/ark:/65665/3f0a81f9e-fd93-4651-b95b-78dc1d4a5c4e) | Scrophulariaceae | *Lindernia* | *dubia* | MF349709 | MF349850 | MF348458 | MF348918 |
| AD7LN15 | [Faulconer 55](http://n2t.net/ark:/65665/3b7932bcc-4d4a-4489-8d01-e050c968872d) | Scrophulariaceae | *Mazus* | *reptans* | MF349419 | MF349947 | MF348751 | MF349124 |
| AD5JQ61 | [Kelloff 1600](http://n2t.net/ark:/65665/3c0e53cff-283e-4053-ad3c-1597da14d318) | Scrophulariaceae | *Penstemon* | *australis* | MF349572 | MF349848 | MF348602 | MF349013 |
| AD5JQ24 | [Van Neste 546](http://n2t.net/ark:/65665/359ed61a3-e0cf-4b4e-b646-14406a3e6d4a) | Scrophulariaceae | *Russelia* | *equisetiformis* | MF349592 | MF350075 | MF348580 | MF348996 |
| AD7LH40 | [Van Neste 764](http://n2t.net/ark:/65665/33360d1e5-57c1-43e9-b94c-cfcf0e4667cf) | Scrophulariaceae | *Verbascum* | *thapsus* | MF349640 | MF350112 | MF348529 | MF348965 |
| AD0EE76 | [Kelloff 1583](http://n2t.net/ark:/65665/30fae972a-9638-44f9-93d2-260edb4ffb38) | Smilacaceae | *Smilax* | *rotundifolia* | MF349230 | MF350285 | MF348346 |  |
| AD7LN85 | [Van Neste 704](http://n2t.net/ark:/65665/3241edb56-4d5e-4cd2-9173-c8fff75ba33b) | Solanaceae | *Atropa* | *belladonna* | MF349619 | MF350095 | MF348553 | MF348980 |
| AD7LI38 | [Van Neste 813](http://n2t.net/ark:/65665/37c5196af-b5c7-440e-a7f2-4880373dc67a) | Solanaceae | *Cestrum* | *nocturnum* | MF349675 | MF350140 | MF348491 | MF348944 |
| AD3HJ94 | [Van Neste 388](http://n2t.net/ark:/65665/3df80ac02-8d10-4d52-993d-89bf9eb61c89) | Solanaceae | *Datura* | *stramonium* | MF349547 | MF350041 | MF348633 | MF349040 |
| AG2KL13 | [Sedaghatpour 2016-60](http://n2t.net/ark:/65665/398138bce-4fa8-4bef-9f8c-ef905f8d427e) | Solanaceae | *Juanulloa* | *aurantiaca* | MF349363 | MF349895 | MF348797 | MF349165 |
| AD5JS83 | [Van Neste 636](http://n2t.net/ark:/65665/3bccc7281-cfef-4be3-8215-e2bf53657a38) | Solanaceae | *Physalis* | *virginiana* | MF349718 | MF350160 | MF348449 |  |
| AD0EE79 | [Kelloff 1586](http://n2t.net/ark:/65665/37a5cce96-7b7c-409c-97ea-fc57a630491d) | Solanaceae | *Solanum* | *carolinense* | MF349791 | MF350216 | MF348367 | MF348868 |
| AD5JQ54 | [Kelloff 1593](http://n2t.net/ark:/65665/322f30f5c-81e7-4ac2-acea-0477bcd9bd50) | Solanaceae | *Solanum* | *carolinense* | MF349610 | MF350089 | MF348562 |  |
| AD5JS72 | [Van Neste 625](http://n2t.net/ark:/65665/34350ef2e-e2ef-4833-9f92-fd2c1193613d) | Sparganiaceae | *Sparganium* | *americanum* | MF349287 | MF350184 | MF348411 |  |
| AD7LM45 | [Gostel 180](http://n2t.net/ark:/65665/3abf105f9-343f-4050-b933-6cbf78ddbe19) | Stachyuraceae | *Stachyurus* | *praecox* var. *matsuzakii* | MF349440 | MF349966 | MF348730 | MF349105 |
| AD3HJ52 | [Van Neste 346](http://n2t.net/ark:/65665/360071141-8185-4c41-a20a-3e845a7aa4f5) | Staphyleaceae | *Euscaphis* | *japonica* | MF349502 | MF350011 | MF348672 | MF349069 |
| AD7LN87 | [Van Neste 702](http://n2t.net/ark:/65665/35bf9e65f-f244-4dd9-8f2d-df56d8de35bd) | Sterculiaceae | *Abroma* | *augustum* | MF349636 | MF349845 | MF348534 | MF348969 |
| AD0EF74 | [Van Neste 449](http://n2t.net/ark:/65665/3cb1e0410-ae79-4b12-a57b-ba62de3b41c4) | Sterculiaceae | *Theobroma* | *cacao* | MF349816 | MF350235 | MF348333 |  |
| AD5JR11 | [Van Neste 449](http://n2t.net/ark:/65665/3cb1e0410-ae79-4b12-a57b-ba62de3b41c4) | Sterculiaceae | *Theobroma* | *cacao* | MF349604 | MF350085 | MF348567 |  |
| AG2KL23 | [Sedaghatpour 2016-65](http://n2t.net/ark:/65665/307a31495-97de-4013-8d47-05ce4091b9d8) | Strelitziaceae | *Strelitzia* | *reginae* | MF349370 | MF349901 |  |  |
| AD7LH93 | [Van Neste 788](http://n2t.net/ark:/65665/3e0cdd517-874f-43f5-823f-79b062bd6c69) | Styracaceae | *Halesia* | *diptera* | MF349278 |  | MF348500 | MF348948 |
| AD3HL48 | [Van Neste 544](http://n2t.net/ark:/65665/351631f21-2fa4-40fe-aae3-47f43c47879a) | Styracaceae | *Halesia* | *tetraptera* | MF349602 |  | MF348570 | MF348988 |
| AD0EE06 | [Van Neste 296](http://n2t.net/ark:/65665/392170b74-5224-4ee2-b92f-2b0296766835) | Styracaceae | *Pterostyrax* | *hispidus* | MF349768 | MF350195 | MF348399 | MF348883 |
| AD3HJ02 | [Van Neste 296](http://n2t.net/ark:/65665/392170b74-5224-4ee2-b92f-2b0296766835) | Styracaceae | *Pterostyrax* | *hispidus* | MF349467 | MF349991 | MF348702 | MF349085 |
| AD3HK36 | [Van Neste 430](http://n2t.net/ark:/65665/3a50271b0-94fb-42aa-96fe-9d7635406ff4) | Styracaceae | *Styrax* | *americanus* | MF349261 |  | MF348595 | MF349009 |
| AD3HJ62 | [Van Neste 356](http://n2t.net/ark:/65665/3d4a0429a-4b7f-402f-a7ae-266755f78acd) | Styracaceae | *Styrax* | *japonicus* | MF349501 |  | MF348673 | MF349070 |
| AD3HJ28 | [Van Neste 322](http://n2t.net/ark:/65665/3a98fd229-7b73-4830-94da-7c5346dc036c) | Styracaceae | *Styrax* | *obassia* | MF349473 |  | MF348698 | MF349081 |
| AD3HJ32 | [Van Neste 326](http://n2t.net/ark:/65665/32701a73d-36b3-47c3-ba06-00673b42b305) | Symplocaceae | *Symplocos* | *paniculata* | MF349525 | MF350027 | MF348653 | MF349056 |
| AB6QQ31 | [Van Neste 132](http://n2t.net/ark:/65665/3d46be058-45ac-4f85-9b2e-1864b965e1a6) | Taxaceae | *Cephalotaxus* | *fortunei* | MF349753 |  | MF348414 | MF348890 |
| AD7LN03 | [Faulconer 49](http://n2t.net/ark:/65665/355564a5a-e676-432b-834c-f8b8110c6aea) | Taxaceae | *Taxus* | *wellichiana* | MF349403 |  | MF348769 | MF349138 |
| AD5JP81 | [Kelloff 1598](http://n2t.net/ark:/65665/38e9fd50a-9c36-42b4-9a1a-8b1df1bae575) | Tetrachondraceae | *Polypremum* | *procumbens* | MF349783 | MF350209 | MF348376 | MF348875 |
| AB6QQ27 | [Van Neste 128](http://n2t.net/ark:/65665/3739969c4-9d0c-4e90-9206-a5ceb1cf4d9b) | Theaceae | *Camellia* | *japonica* | MF349713 | MF349836 | MF348454 |  |
| AD5JP53 | [Van Neste 555](http://n2t.net/ark:/65665/39dad1a6e-0cad-4e5b-a41d-3dd964a684f2) | Theaceae | *Franklinia* | *alatamaha* | MF349693 | MF349841 | MF348476 | MF348933 |
| AD7LN59 | [Van Neste 734](http://n2t.net/ark:/65665/339f7de7e-1a70-470b-a303-f67a81e66dab) | Theaceae | *Stewartia* | *pseudocamellia* | MF349661 | MF350128 | MF348507 | MF348952 |
| AD5JS77 | [Van Neste 630](http://n2t.net/ark:/65665/3ca5b6ac1-9da3-40f0-b1d6-31a40701ec24) | Thelypteridaceae | *Thelypteris* | *hexagonoptera* | MF349728 |  |  |  |
| AD5JS47 | [Van Neste 600](http://n2t.net/ark:/65665/31fa3b9b3-53e3-4590-9d1a-5948ea92ddea) | Thelypteridaceae | *Thelypteris* | *noveboracensis* | MF349500 |  | MF348674 |  |
| AD5JT39 | [Van Neste 667](http://n2t.net/ark:/65665/39fde477f-fa86-4f49-9ea3-a3ec097b2252) | Thymelaeaceae | *Edgeworthia* | *papyrifera* | MF349726 | MF350165 | MF348440 | MF348910 |
| AD7LI22 | [Van Neste 797](http://n2t.net/ark:/65665/354d2bb4f-a488-412e-8fa4-a0c8e9c79134) | Tiliaceae | *Sparmannia* | *africana* | MF349676 | MF350141 | MF348490 | MF348943 |
| AB6QQ28 | [Van Neste 129](http://n2t.net/ark:/65665/335318336-dab9-4f7d-8522-f5df22e90cfe) | Trochodendraceae | *Trochodendron* | *aralioides* | MF349702 | MF350154 |  | MF348926 |
| AD0EF14 | [Van Neste 414](http://n2t.net/ark:/65665/386758729-d8ca-4f13-b7ac-ef208fc5d867) | Typhaceae | *Typha* | *latifolia* | MF349809 | MF350230 |  |  |
| AD0EF14 | [Van Neste 414](http://n2t.net/ark:/65665/386758729-d8ca-4f13-b7ac-ef208fc5d867) | Typhaceae | *Typha* | *latifolia* |  |  | MF348341 |  |
| AD7LM73 | [Vo 74](http://n2t.net/ark:/65665/322029323-df25-45d8-b285-b332dd0cc866) | Ulmaceae | *Ulmus* | *americana* | MF349395 | MF349922 | MF348777 |  |
| AD7LM61 | [Sedaghatpour 2016-50](http://n2t.net/ark:/65665/38deefb5d-57d2-4cdd-a313-1e9bcc546c28) | Ulmaceae | *Ulmus* | *Chenmuoi* | MF349450 | MF349975 | MF348719 |  |
| AD5JS44 | [Van Neste 597](http://n2t.net/ark:/65665/3531d42b7-63e8-4e5e-86d4-e1a14afa7c20) | Urticaceae | *Boehmeria* | *cylindrica* | MF349739 |  |  | MF348900 |
| AD5JT02 | [Van Neste 692](http://n2t.net/ark:/65665/33d336b54-ffdc-4d6e-93e8-08513937facc) | Urticaceae | *Boehmeria* | *nivea* | MF349629 | MF350103 | MF348543 | MF348975 |
| AD5JT12 | [Van Neste 639](http://n2t.net/ark:/65665/3c83b43dd-96ff-4315-8899-059588559dcb) | Valerianaceae | *Centranthus* | *ruber* | MF349697 | MF350151 | MF348471 | MF348930 |
| AD7LN80 | [Van Neste 709](http://n2t.net/ark:/65665/39a492d94-2cc4-4e74-a03c-11f475e8ef46) | Valerianaceae | *Valeriana* | *officinalis* | MF349672 | MF350137 | MF348494 |  |
| AD5JP67 | [Van Neste 569](http://n2t.net/ark:/65665/3e1a96389-3e84-425a-ada5-1530bbd25420) | Verbenaceae | *Aloysia* | *virgata* | MF349731 | MF350169 | MF348435 | MF348906 |
| AD3HI39 | [Van Neste 233](http://n2t.net/ark:/65665/3b2b563da-656a-4ed6-b4a4-b0b513649e5b) | Verbenaceae | *Phyla* | *dulcis* | MF349497 | MF350009 | MF348677 | MF349072 |
| AD3HK17 | [Van Neste 411](http://n2t.net/ark:/65665/3e58b41ff-cdec-463b-bb01-20a9438c86cf) | Verbenaceae | *Phyla* | *lanceolata* | MF349578 | MF350066 | MF348594 |  |
| AG2KK31 | [Gostel 209](http://n2t.net/ark:/65665/3b6b4d7c3-ac57-490d-917e-509d8a6697e8) | Verbenaceae | *Stachytarpheta* | *mutabilis* var. *violacea* | MF349388 | MF349915 | MF348783 |  |
| AD5JT22 | [Van Neste 649](http://n2t.net/ark:/65665/300c139af-0948-46fb-be0f-509b1135ec4a) | Verbenaceae | *Verbena* | *bonariensis* | MF349727 | MF350166 | MF348439 | MF348909 |
| AD0EF21 | [Van Neste 392](http://n2t.net/ark:/65665/3e976d5ca-4f02-4484-a5a1-d6866c609cf3) | Verbenaceae | *Verbena* | *urticifolia* | MF349787 | MF350211 |  | MF348872 |
| AD0EF21 | [Van Neste 392](http://n2t.net/ark:/65665/3e976d5ca-4f02-4484-a5a1-d6866c609cf3) | Verbenaceae | *Verbena* | *urticifolia* |  |  | MF348373 |  |
| AD5JS66 | [Van Neste 619](http://n2t.net/ark:/65665/364e6c607-893f-470b-8fd1-ee5f92ddeca6) | Verbenaceae | *Verbena* | *urticifolia* |  |  | MF348429 | MF348901 |
| AG2KK53 | [Vo 86](http://n2t.net/ark:/65665/3a137d32c-89c7-49d5-bb42-959b16b5d11d) | Violaceae | *Viola* | *labradorica* | MF349366 | MF350252 |  | MF349163 |
| AD7LH14 | [Van Neste 737](http://n2t.net/ark:/65665/3636e0b4a-4f3c-4607-9d15-5c86051c1ee2) | Vitaceae | *Ampelopsis* | *brevipedunculata* | MF349642 | MF350114 | MF348527 |  |
| AD7LN68 | [Van Neste 721](http://n2t.net/ark:/65665/3126120a1-4e57-4d8c-a711-e4ba96d82419) | Vitaceae | *Ampelopsis* | *cordata* | MF349618 | MF350094 | MF348554 |  |
| AD7LH31 | [Van Neste 754](http://n2t.net/ark:/65665/359087404-1d89-4803-900c-4c0258148db6) | Vitaceae | *Ampelopsis* | *glandulosa* | MF349641 | MF350113 | MF348528 |  |
| AB6QQ05 | [Van Neste 105](http://n2t.net/ark:/65665/32a7c03fe-59ef-46ae-99d8-edafa7f43b9a) | Vitaceae | *Cissus* | *adenopoda* | MF349743 | MF350177 | MF348423 |  |
| AD5JR04 | [Van Neste 590](http://n2t.net/ark:/65665/33d38e3f6-2cbd-4d71-9736-40940d19f42a) | Vitaceae | *Cissus* | *rotundifolia* | MF349686 | MF350187 | MF348409 |  |
| AD5JQ94 | [Van Neste 580](http://n2t.net/ark:/65665/3a9c90057-9736-4461-adc8-fe2ae33bab7a) | Vitaceae | *Parthenocissus* | *heptaphylla* | MF349286 | MF350168 | MF348436 |  |
| AD7LH95 | [Van Neste 790](http://n2t.net/ark:/65665/37f5aaac4-0ce6-48cc-979f-b4386b38cae0) | Vitaceae | *Parthenocissus* | *quinquefolia* | MF349648 | MF350119 | MF348521 |  |
| AD3HJ77 | [Van Neste 371](http://n2t.net/ark:/65665/37f343925-cbec-43ad-8ef2-34d208ea33b5) | Vitaceae | *Vitis* | *cinerea* | MF349248 | MF349990 | MF348705 |  |
| AD5JQ55 | [Kelloff 1594](http://n2t.net/ark:/65665/31a286567-68ff-4f60-b8e5-dbb58cef024d) | Vitaceae | *Vitis* | *rotundifolia* | MF349601 | MF350082 | MF348572 |  |
| AD5JS55 | [Van Neste 608](http://n2t.net/ark:/65665/3c3dec7b4-1eb8-44dd-a808-cc6ff06b22cb) | Xyridaceae | *Xyris* | *torta* | MF349738 |  | MF348428 |  |
| AG2KL07 | [Sedaghatpour 2016-57](http://n2t.net/ark:/65665/36a1e76c7-3f84-49bd-8530-9250fbc7f818) | Zamiaceae | *Dioon* | *edule* | MF349313 |  | MF348837 |  |
| AG2KM60 | [Marcelli 34](http://n2t.net/ark:/65665/3cae88bef-167a-4dd6-a6bd-3b2bd21e2393) | Zingiberaceae | *Alpinia* | spp. | MF349223 |  |  | MF349156 |
| AG2KL33 | [Sedaghatpour 2016-70](http://n2t.net/ark:/65665/32dd2bead-bf3b-48fa-9f98-c5de6180ecf9) | Zingiberaceae | *Curcuma* | *cordata* | MF349352 | MF349887 | MF348806 |  |
| AD5JQ88 | [Van Neste 574](http://n2t.net/ark:/65665/3baaa22d6-c6c2-4872-9c05-f5a33deb8898) | Zingiberaceae | *Globba* | *lithophytica* | MF349692 |  | MF348477 | MF348934 |
| AB6QQ12 | [Van Neste 112](http://n2t.net/ark:/65665/3aa1e4418-adfb-4d01-ad4e-534d768a3efb) | Zingiberaceae | *Globba* | *patens* | MF349714 |  | MF348453 | MF348917 |
| AD5JQ89 | [Van Neste 575](http://n2t.net/ark:/65665/3865f0edf-dee8-406e-8acc-d8ca473ba0ab) | Zingiberaceae | *Hedychium* | *glabrum* | MF349211 |  |  | MF348886 |
| AD3HI07 | [Van Neste 201](http://n2t.net/ark:/65665/313526910-2659-4699-ad2c-cd1d82a00633) | Zingiberaceae | *Hedychium* | *longicornutum* |  |  |  | MF349051 |
| AD5JQ90 | [Van Neste 576](http://n2t.net/ark:/65665/3187ea509-874f-4055-bc73-0b5b0cc1782d) | Zingiberaceae | *Kaempferia* | *elegans* | MF349751 |  |  | MF348892 |
| AG2KL31 | [Sedaghatpour 2016-69](http://n2t.net/ark:/65665/32330446e-0d9e-4727-bb84-241b85c9baf9) | Zingiberaceae | *Siphonochilus* | *kirkii* | MF349362 |  |  | MF349166 |
| AG2KL09 | [Sedaghatpour 2016-58](http://n2t.net/ark:/65665/38c2a4153-a944-4ccb-9a28-b7c5bc50e68f) | Zingiberaceae | *Smithatris* | *myanmarensis* | MF349384 | MF349913 | MF348784 | MF349152 |
